# Gut microbiome alterations are sex-dependently associated with brain abnormalities in a mouse model of Neurofibromatosis type I

**DOI:** 10.1038/s41380-026-03609-0

**Published:** 2026-04-27

**Authors:** Sonali N. Reisinger, Geraldine Kong, Nicholas van de Garde, Asim Muhammad, Pranav Adithya, Da Lu, Pamudika Kiridena, Carolina Gubert, Gabriel Dabscheck, Jonathan M. Payne, Anthony J. Hannan

**Affiliations:** 1 Florey Institute of Neuroscience and Mental Health, Parkville, VIC Australia; 2 Peter Doherty Institute of Infection and Immunity, Department of Microbiology and Immunology, University of Melbourne, Parkville, VIC Australia; 3 Centre for Pathogen Genomics Innovation Hub, Department of Microbiology and Immunology, University of Melbourne, Parkville, VIC Australia; 4 Florey Department of Neuroscience and Mental Health, University of Melbourne, Parkville, VIC Australia; 5 Royal Children’s Hospital Melbourne, Parkville, VIC Australia; 6 Murdoch Children’s Research Institute, Parkville, VIC Australia; 7 Department of Paediatrics, University of Melbourne, Parkville, VIC Australia; 8 Department of Anatomy and Physiology, University of Melbourne, Parkville, VIC Australia

**Keywords:** Neuroscience, Autism spectrum disorders, Diagnostic markers, ADHD, Physiology

## Abstract

Neurofibromatosis type 1 (NF1) is a genetic condition presenting with variable symptomatology, however most individuals will demonstrate cognitive and behavioural difficulties, including autism. Using a heterozygous germline knockout mouse model of NF1 (*Nf1 +/-*), we performed in-depth behavioural evaluations encompassing learning and memory, stereotypy, social interaction, anxiety- and depression-like behaviour. Anatomical and functional studies of the brain and gastrointestinal tract were followed by the first investigation of gut microbiota composition (via full-length 16S rRNA sequencing) in a *Nf1 +/-* mouse model. The cognitive and autism-like behavioural phenotype seen in *Nf1 +/-* mice was accompanied by a striking increase in relative brain size which is highly relevant to clinical NF1. Furthermore, brain size was correlated with behaviour, supporting a potential mechanistic link. *Nf1 +/-* mice showed significant alterations in gut microbiota composition vs. *Nf1 +/+* wild-type controls, with males additionally showing significant changes to species abundance of the *Clostridium* and *Blautia* genera, and the *Lachnospiraceae* family, findings which partially overlap with those in preclinical and clinical autism. Composition of associated functional pathways was not globally altered, however *+/-* mice showed significant changes in a pyrimidine deoxynucleotide biosynthesis pathway. In male *Nf1 +/-* mice, we also identified a genotype-specific host-microbial signature, pointing towards a mechanistic link between gut microbiome composition and brain size. These findings significantly expand our understanding of brain and behavioural abnormalities in this preclinical model of NF1 and, importantly, have uncovered the gut microbiome as a highly promising new area of research and a potential therapeutic target for these symptom clusters.

## Introduction

Neurofibromatosis type 1 (NF1) is an autosomal dominant genetic disorder affecting heterozygous carriers of a mutant copy of the neurofibromin gene (*NF1*), accounting for around 1 in 3000 births [1, 2]. As a negative regulator of the Ras-MAPK [3] and PI3K-Akt-mTOR pathways [4], neurofibromin plays a central role across many tissues in the regulation of cell proliferation and is considered a tumour-suppressing protein. Notably, individuals with NF1 show a range of multisystemic symptoms of varying severity. In somatic tissues, neurofibromin deficiency is thought to be compounded by secondary loss of heterozygosity, leading to uncontrolled cell proliferation. This can lead to cell malformations including tumours across many tissues of the body, the most common of which are plexiform neurofibromas (affecting nerve sheaths), Lisch nodules (affecting the iris) and café-au-lait macules (affecting epidermal melanocytes). Other common symptoms include bone abnormalities and hypertension. While mostly benign, arising tumours can lead to chronic pain, cosmetic disfigurement, may impede the normal growth of critical structures (e.g. airway or spinal cord), and can reduce overall functional outcomes. Furthermore, malignancies may develop in a significant subset of individuals with NF1, greatly reducing life expectancy [5].

Neurofibromin is widely expressed throughout the brain across early development and adulthood [6, 7]. The prevailing view is that neurofibromin protein deficiency leads to neurodevelopmental and neurophysiological abnormalities that contribute to the cognitive, behavioural, and psychiatric symptom complexes seen in many individuals with NF1 [8, 9]. A downward shift in intellectual functioning is a robust finding and specific deficits in executive function, attention and visuospatial perception have been widely reported [10, 11]. These cognitive complications contribute to elevated autistic traits [12] and attention deficit hyperactivity disorder (ADHD) symptoms [13], with approximately 25% of children with NF1 meeting diagnostic threshold for autism [14] and 38% for ADHD [15]. Additionally, a majority of individuals with NF1 present with learning difficulties [16, 17]. Rates of depression and anxiety are also significantly higher in those with NF1 than in the general population, although exact estimates vary [18, 19]. Despite the negative impact on quality of life [20], no targeted treatments for cognitive deficits and psychiatric symptoms in NF1 are currently available. Heterozygous *Nf1 +/-* mice have been used to model cognitive and behavioural symptoms in this condition [21]. This preclinical model does not develop malignancies until an advanced age [22], thus avoiding the confounding factor of tumour formation on relevant outcomes. Research using the *Nf1 +/-* mouse has repeatedly revealed deficits in spatial learning [21, 23–26] and several other cognitive domains [26–29], although there have also been a number of inconsistent results [26, 30, 31]. In addition, behavioural alterations highly relevant to the autism-associated symptoms seen in individuals with NF1 have been reported in *+/-* mice, including significant changes in early ultrasonic vocalization [32], suggesting communication deficits, as well as impairments in social recognition [25, 28, 32].

Other phenotypes in *Nf1 +/-* mice pertinent to NF1 clinical presentation include morphometric MRI findings of several brain regions [25]. In humans, enlarged brains and brain subregions have been reported consistently [33–39], and some studies have reported a correlation between these structural abnormalities and the severity of specific neuropsychological traits [34, 36]. Neurodevelopmental disorders such as autism and other syndromes that have overlapping symptoms with NF1—such as fragile X syndrome—are also associated with increased brain size [39–42], which points toward a potential significance of this trait in the shared pathophysiology. In mice, increased prefrontal cortex volumes in *+/-* were found to be inversely correlated with social recognition performance, while striatum volumes inversely predicted spatial learning performance for a subgroup of *+/-* mice [25]. While these associations do not constitute proof of a causal role for brain size in the emergence of these behavioural traits, they provide convincing support for further investigations into the role of altered brain structure in NF1 neurobehavioural phenotypes.

The study of the gut microbiome has revealed altered configurations in a number of major psychiatric disorders as a novel, and targetable, aspect of pathophysiology [43–45]—including in autism [46, 47]. Surprisingly, there have been no studies to date examining the gut microbiota composition of individuals with NF1, nor of heterozygous *Nf1 +/-* preclinical models of this disorder. This area thus represents an entirely novel sub-field of research with considerable potential for the development of new treatment strategies for individuals with NF1, which need to be urgently explored.

Here, we employed the *Nf1 +/-* mouse model to systematically evaluate a wide range of behaviours relevant to all brain-related symptoms seen in NF1, including the domains of cognition, anxiety and depression, as well as autism- and ADHD-relevant behaviours. As NF1 affects both males and females equally, with some divergence in symptomatology [48], male and female mice were included in all experiments. We report neurofibromin deficiency-induced changes in clinically relevant brain anatomical measures, combined cognitive and autism-like behavioural measures, reduced gut inflammation and, for the first time, show baseline gut microbiota compositional changes in a preclinical model of NF1. These microbiota abnormalities are more pronounced in males, and partially resemble gut microbiota alterations associated with autism. Furthermore, while predicted specific functional pathways impacted by this change were limited, we uncovered that in males, brain weight, stool softness and several microbial species covaried and collectively allowed discrimination between genotypes. Taken together, these findings uncover a novel area of research and first mechanistic insights into a potential dysregulation of the microbiota-gut-brain axis in NF1. In the quest for new treatments targeting NF1 cognitive and behavioural symptoms, our findings provide a strong basis for urgently prioritising the investigation of the gut microbiome in individuals with NF1, and its role in the pathophysiology of this common genetic condition.

## Materials and methods

### Animals

Founder pairs of *Nf1 +/-* mice (B6.129S2-Nf1tm1Tyj/J; originally generated by Jacks and colleagues [22]) were obtained from The Jackson Laboratory (Bar Harbor, ME, USA) and maintained on a C57BL6/J background. To minimise potentially confounding effects of altered maternal care behaviour provided by *Nf1 +/-* mothers, only Nf1 *+/+* females were bred with *Nf1 +/-* males to generate *+/+* and *+/-* offspring. Mice were toe clipped in the first postnatal week and biopsies were sent to Transnetyx, Inc. (Memphis, TN, USA) for genotyping of the *Nf1* locus. All experiments were conducted with the experimenter blinded to the genotypes. Mice were housed in groups of 2-5 per cage, separated by sex, under specific pathogen free (SPF) conditions in open-top cages with standard wood-chip bedding (Tapvei Estonia Oü, Harjumaa, Estonia) and with *ad libitum* access to water and standard chow (Ridley, Melbourne, Australia). For cohorts 1 and 2, cages contained both *+/-* and *+/+* mice, randomly mixed across different litters. In cohort 3, for gut function and microbiota studies, genotypes were separated to ensure gut microbiota would not be shared between mice of different genotypes within the same cage, but litters were randomised across cages. Housing rooms were on a 12:12 light-dark cycle (lights on: 8:00) and temperature was kept between 19-23 °C. Body weight was recorded once per week. All animal experiments were performed in accordance with the Australian Code for the Care and Use of Animals for Scientific Purposes, with experiments vetted and approved by the Animal Ethics Committee of the Florey Institute of Neuroscience and Mental Health (project #22-017).

### Behavioural analysis

**Fig. 1:**
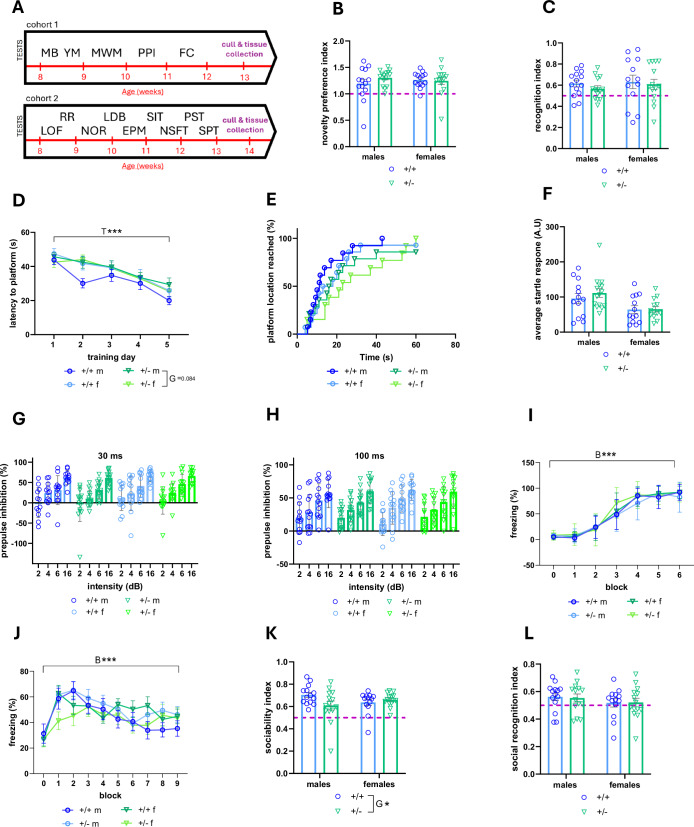
Cognitive and social behaviours in *Nf1***+**/- mice. (**A**) Timelines of experimental procedures in cohorts 1 and 2. Male and female *+/+* and *+/-* mice were subjected to extensive behavioural testing for in-depth phenotypic characterisation relevant to all brain-related symptom complexes experienced by NF1 patients. Cognitive behaviour was evaluated in the (**B**) Y maze test, the (**C**) novel object recognition test, and the Morris water maze (**D, E**). Both *+/+* and *+/-* mice showed normal preference for exploring the novel arm (**B**) and investigating a novel object (**C**), suggesting intact short-term memory. Nf1-deficient mice showed a trend toward (**D**) longer latencies to reach a hidden platform (s) across five training days in the MWM (Genotype: p = 0.084; Training day: p < 0.001***). On the (**E**) probe test day, where the platform is removed, no differences between groups emerged. Average startle response to an auditory stimulus in the prepulse inhibition test (**F**) did not differ between groups. Sensorimotor gating was also unaffected in *+/-* mice, as neither a (**G**) 30 ms or (**H**) 100 ms prepulse revealed any significant differences in behaviour (Intensity: p < 0.001*** for 30 ms and 100 ms prepulse). Freezing response in (**I**) fear conditioning training (Block: p < 0.001***) and the (**J**) fear extinction learning task did not differ between groups, indicating similar levels of cued associative learning. In the social interaction test, (**K**) sociability was first evaluated by measuring the preference for interaction with a novel mouse over an empty compartment of the chamber, where *Nf1 +/-* showed a significant reduction in sociability index (Genotype: p = 0.0361*; Genotype x Sex: p = 0.0651). The (**L**) social recognition index was not significantly different, since when given a choice between a novel and a familiar mouse, on average mice across experimental groups interacted more with the novel mouse. Individual values are shown with mean and SEM error bars (**B**, **C,** **F-H,** **K, L**), mean values are shown with SEM error bars (**D, I, J**) or individual values are shown (**E**). Dotted line indicates chance level performance (**B, C, K, L**). * denotes p < 0.05; *** denotes p < 0.001. **G**: main effect of genotype; S: main effect of sex; T: main effect of training day; B: main effect of time block. MB: marble burying, MWM: Morris water maze, YM: Y maze; PPI: prepulse inhibition, FC: fear conditioning, LOF: large open field, EPM: elevated plus maze, NSFT: novelty-suppressed feeding test, LDB: light-dark box, SIT: social interaction test, NOR: novel object recognition, RR: Rotarod, PST: Porsolt swim test, SPT: saccharin preference test.

Behavioural evaluations were performed starting at 8 weeks old (see Fig. 1A for a timeline of procedures in cohorts 1 and 2). Prior to all behavioural tests, mice were transported to the experimental room and allowed to habituate for 30-60 min before testing. Mice were allowed to rest for a minimum of 24 h between tests, with > 48 h rest after the most stressful tests (Morris water maze, fear conditioning, Porsolt swim test) or those involving fasting (novelty-suppressed feeding, gut transit time). Tests were performed in ascending order of stressfulness, so as to not impact performance in subsequent tests, and were split between cohorts to maintain similar levels of impact (with cohort 1 undergoing fewer, but longer and individually more stressful tests compared to cohort 2), as depicted in Fig. 1A timelines. After the end of behaviour experiments, 1 week of rest was allowed before tissue collection at 13-14 weeks of age.

#### Large open field

As previously published, mice were placed into the corner of a square arena (1 m x 1 m x 0.2 m) with opaque walls, and the centre illuminated to 1000 lx [49]. Their movements were tracked using an overhead mounted camera and TopScan Lite software (CleverSys, Reston, VA, USA) for 10 min. Total distance travelled (cm) was measured to evaluate spontaneous locomotor activity, and time spent in the centre of the arena (%) was analysed as measures of anxiety-like behaviour, as the exposed, brightly lit centre is considered aversive to mice [50].

#### Light-dark box

Apparatus (Med Associates Inc., Fairfax, VT, USA) consisted of a clear Plexiglas box (ENV-510, 27 cm×27 cm x 20.5 cm, Med Associates Inc.) fitted with infrared beam arrays (ENV-256, Med Associates Inc.) to track motion. Opaque inserts covering half of the area (ENV-511, 27 cm×13.5 cm×20.5 cm, Med Associates Inc.), fitted with a small opening in the centre of the arena, and a trap door at the top, were used as the dark compartment. Illuminance at the centre of the light zone was set to 750 lx. Mice were placed into the dark compartment and allowed to explore the dark and aversive light zones for 10 min [51]. Total distance travelled (cm), time spent in light zone (%) were analysed, with the latter indicating levels of anxiety-like behaviour as the brightly lit zone is considered aversive to mice [52].

#### Elevated plus maze

Mice were placed into the centre of a plus-shaped maze elevated ~40 cm off the ground, consisting of two opposing ‘open’ arms, and two opposing ‘closed’ arms (30 cm×5 cm, with 15 cm high opaque walls on the closed arms) for a 5-min trial, as previously reported [53]. Mice were tracked using an overhead camera and Topscan Lite software (CleverSys). Time spent in the aversive open arms (%) was analysed as measures of anxiety-like behaviour [54].

#### Novelty-suppressed feeding

In order to measure levels of depression-related anxiety, hyponeophagia was evaluated [55, 56]. Mice were weighed and food-restricted 24 h prior to testing. A pellet of standard chow (Ridley) was attached to a circle of filter paper (diameter ~ 8 cm), placed in the centre of a high-walled open field (60 cm×60 cm x 50 cm) with wood-chip bedding (Tapvei Estonia Oü) covering the floor. Mice were weighed, then placed in one corner of the arena and the latency (s) to feed on the pellet was recorded, observable as the mouse sitting on its hind legs, holding the food pellet with both hands and biting into it. Once the first bite was taken, mice were immediately removed from the arena and single housed in a holding cage for 5 min with a pre-weighed pellet of standard chow (Ridley). At 5 min, the pellet was removed and the amount consumed was determined in order to evaluate differences in hunger between mice resulting from the 24 h fast.

#### Porsolt swim test

As previously published, mice were placed in a 2 L glass beaker filled with roughly 1250 mL of 24 °C ( ± 1 °C) water for 6 min [57]. The final 4 min of the test were automatically analysed for floating or immobility behaviour (%) using the DepressionScan software suite (Cleversys) as a measure of depressive-like behaviour. Sensitivity settings for automatic movement analysis were determined by an experienced experimenter, with immobility defined as the absence of swimming movements except for those required to stay at the surface.

#### Saccharin preference test

Mice were single housed and habituated to drinking from two bottles equipped with sipper tubes, both filled with standard filtered tap water, for 24 h [58]. Following this period, mice were given the choice between a bottle with water and a sweet 0.1 % saccharin solution (#S1002, Sigma-Aldrich, St Louis, MO, USA) for 48 h. Bottles were weighed at t = 0 h, t = 24 h, and t = 48 h to determine water and saccharin consumption per 24 h period. To avoid effects of side preference, water and saccharin bottles were placed on alternating sides, and sides were switched at t = 24 h. Saccharin preference (%) was calculated and used as a measure of anhedonia according to the formula:

$${SP}=\frac{{saccharin}\,{solution}\,{consumed}\,\left(g\right)}{{water}\,{consumed}\,\left(g\right)+{saccharin}\,{solution}\,{consumed}\,\left(g\right)}\times 100$$

#### Marble burying

Mice were placed in a transparent polycarbonate box (37.5 cm×20 cm x 20 cm) with approximately 5 cm of standard wood-chip bedding (Tapvei Estonia Oü) and 24 glass marbles (1.5 cm diameter) evenly spaced at a distance of ~ 4 cm from the walls of the box. After 30 min, the number of marbles buried were counted as a measure of a neophobic anxiety-like response or compulsive behavior [59, 60]. A marble was counted as ‘buried’ when it was at least two-thirds covered by bedding after 30 min.

#### Novel object recognition

As previously published [49], mice were placed in a square arena (50 cm×50 cm x 50 cm) and their movements were recorded with an overhead mounted camera and tracked using TopScan Lite software (CleverSys). Mice were allowed to explore the empty arena for 5 min to habituate to the apparatus. 24 h later, mice were returned to the same arena for 10 min, with two identical objects, either 250 mL Falcon cell culture flasks filled with sand, or multicoloured LEGO DUPLO towers (6.5 cm × 6.5 cm × 16.5 cm; Lego, Billund, Denmark) [61]. 1 h later, mice were returned to the arena for 10 min for another trial, with one object swapped out for the alternative type, resulting in mice being exposed to two different objects. Mice were removed from the analysis if they perched on top of the objects. As mice typically prefer to investigate a novel object over a familiar one, a recognition index was calculated as a measure of short-term memory according to the formula:

$${RI}=\frac{{duration}\,{interacting}\,{with}\,{novel}\,{object}\,\left(s\right)}{{duration}\,{interacting}\,{with}\,{novel}\,{object}\,\left(s\right)+{duration}\,{interacting}\,{with}\,{familiar}\,{object}\,\left(s\right)}$$

#### Y maze

The apparatus comprised three arms (38 cm×7.5 cm; 13 cm high walls) at 120° angles to each other, with distinct black and white pattern cues affixed at the end of each arm: the home arm, familiar arm, and novel arm. As previously published, in trial 1, mice were placed in the home arm and allowed to explore the home and familiar arms for 10 min, with a door blocking entry to the novel arm [62]. After a 1 h inter-trial interval, mice were returned to the home arm, with both the familiar and novel arms available to be explored, for 5 min. Mice were tracked using an overhead camera and Topscan Lite software (CleverSys). As mice typically prefer to investigate a novel area over a familiar one, a novelty preference index was calculated as a measure of short-term memory and spatial learning according to the formula:

$${NPI}=\frac{{duration}\,{in}\,{novel}\,{arm}\,\left(s\right)}{{average}\left({duration}\,{in}\,{novel}\,{arm}\,\left(s\right)+{duration}\,{in}\,{familiar}\,{arm}\,\left(s\right)\right)}$$

#### 3-chamber social interaction test

Apparatus consisted of a transparent Plexiglas box (San Diego Instruments, Inc., CA, USA; 40 cm × 37.5 cm × 10 cm) separated into three chambers by transparent walls with central openings. The two outer chambers (16 cm × 37.5 cm × 10 cm) each contained a metal cage (15.5 cm × 10.5 cm × 9.8 cm) to one side. The test comprised three trials performed sequentially, with the experimental mouse tracked using an overhead camera and Topscan Lite software (CleverSys) [63, 64]. Experimental mice were removed from the arena between trials. In the first round, mice were placed in the central chamber (7.5 cm × 37.5 cm × 10 cm) and allowed to explore for 5 min to habituate to the empty arena. In the second, a sex- and age-matched ‘guest’ mouse was placed into the cage in one of the outer chambers, keeping the other one empty. The experimental mouse was returned to the central chamber and allowed to explore the maze for 5 min. Sociability was evaluated by calculating an index according to the formula:

$${SI}=\frac{{duration}\,{in}\,{chamber}\,{with}\,{guest}\,{mouse}\,\left(s\right)}{{duration}\,{in}\,{chamber}\,{with}\,{guest}\,{mouse}\,\left(s\right)+{duration}\,{in}\,{empty}\,{chamber}\,\left(s\right)}$$

In the third trial, another sex- and age-matched ‘stranger’ mouse was placed into the second cage, while the ‘guest’ mouse (now called ‘familiar’) remained in its cage. The experimental mouse was again returned to the central chamber and allowed to explore for another 5 min. A mouse typically seeks social novelty, so social memory was assessed by recording the latency to interact with the stranger mouse and with a social recognition index calculated according to the formula:

$${SI}=\frac{{duration}\,{in}\,{chamber}\,{with}\,{stranger}\,{mouse}\,\left(s\right)}{{duration}\,{in}\,{chamber}\,{with}\,{stranger}\,{mouse}\,\left(s\right)+{duration}\,{in}\,{familiar}\,{chamber}\,\left(s\right)}$$

#### Morris water maze

Spatial learning and recall were measured in the Morris water maze [65]. A round pool (diameter 1.2 m) was filled with water (25 °C ± 1 °C) mixed with ~ 50 mL of non-toxic white paint to produce an opaque liquid. Visual cues were placed at the north, east, south and west edges of the pool to aid orientation. Mice were trained to find a platform submerged ~ 0.5 cm below the surface in the south-east quadrant of the pool, for 5 days (4 trials per day, per mouse). At the beginning of each trial, mice were released at different locations in the pool and were allowed to search for the platform for 1 min. If not located, mice were led to the platform by the experimenter. After finding the platform, mice were left undisturbed for 30 s. Trials were recorded using an overhead camera and Topscan Lite tracking software (Cleversys). After each trial, mice were gently dried using a towel and placed in a holding cage under a heat lamp for 10-15 min. Following training days, the platform was removed for the probe trial on day 6. Here, mice were released at the north-west edge of the pool and allowed to search for the platform for 1 min, with the latency to reach the former location of the platform analysed as a measure of spatial memory performance [66].

### Prepulse inhibition of acoustic startle

Mice were placed within automated SR-LAB Startle Response System (San Diego Instruments, Inc.) equipment measuring mouse startle response to an acoustic stimulus [67]. The mice were administered a sequence of 104 trials. The first and last eight trials consisted of ‘pulse only’ stimuli of a loud 115 dB for 40 ms (startle tone). The middle 88 trials consisted of a random sequence of 16 ‘pulse only’ startle tones, 8 no-stimulus trials, and 64 prepulse trials. For prepulse trials, a prepulse tone either 30 ms or 100 ms prior to the startle tone at 2, 4, 8 or 16 dB above 70 dB background for 20 ms was played. Prepulse inhibition was calculated for each prepulse tone and delay according to the formula, representing a measure for sensorimotor gating:

$${PPI}=\frac{{median}\,{startle}\,{response}\,{pulse}\,{only}\,{stimuli}-{median}\,{startle}\,{response}\,{prepulse}\,{trials}}{{median}\,{startle}\,{response}\,{of}\,{pulse}\,{only}\,{stimuli}}\times 100$$

#### Fear conditioning and extinction

Fear conditioning and extinction paradigms were performed as previously published [62]. In short, mice were placed into fear conditioning chambers (Med Associates Inc.) controlled via VideoFreeze software (Med Associates Inc.). To create two different environments for conditioning and extinction trials, chambers either contained patterned background walls and paper towel bedding (context A), or no background and standard wood-chip bedding (context B; Tapvei Estonia Oü). Conditioning involved an auditory conditioned stimulus (CS) played for 10 s (80 dB; 5000 Hz) paired with a foot shock unconditioned stimulus (US) for 1 s (0.7 mA). Mice were habituated to the chambers for 2 min while measuring baseline freezing behaviour. Subsequently, mice received six paired CS-US, with the CS co-terminating with the US, and with a 100 s break between CS-US blocks. Time spent freezing (%) during the 9 s before the onset of the foot shock was used to assess the conditioned response to the tone over time. Mice remained in the chamber for 2 min following the last CS-US. Following a 24 h interval, extinction training was performed in a different environmental context (B or A, respectively). Mice were allowed to habituate to the new chamber for 2 min, while baseline freezing was measured. Mice were then presented with 45 CSs without the US, with a 10 s interval between tones. Freezing during the CS tone was recorded, and average freezing across nine blocks of five CSs, was analysed to assess extinction learning over time. Differences in baseline freezing were evaluated in a preliminary analysis and included as a covariate in the statistical model where appropriate.

#### Calculation of integrative z-scores

Integrative z-scores were calculated from relevant measurements and test parameters, in order to detect subtle brain-behavioural phenotypes which are congruent across several tests and measurements, collectively evaluating similar behavioural domains and endophenotypes [68, 69]. Initially, individual z-scores (Z_i_) were calculated for each animal, and each test parameter included in the integrative z-scores, according to the following formula:

$${Z}_{i}=\frac{X-\,\mu }{\sigma }\,$$

where X is the value for a particular parameter and mouse; μ is the control mean for the parameter (calculated separately for each sex); and σ is the standard deviation of the control group for the parameter. For integrative z-scores covering several tests, Z_i_ scores of different parameters of the same behavioural test were first averaged for each mouse, to ensure different tests were weighted equally. The resulting z-scores for each test were averaged for each mouse for each behavioural domain, including cognition, autism-like characteristics, emotionality, and locomotion. Details of parameters and tests included in each integrative z-score are listed in Supplementary table 1. While the majority of integrative z-scores involved summarising behavioural test parameters, the post-mortem brain weight (relative to body weight) was included in the autism-like z-scores, as a measure specifically relevant to the autism endophenotype in NF1 [39, 70].

### Gut health and function

#### Faecal pellet output and faecal water content

At 8 weeks old, mice from cohort 3 were single housed for 1 h in clean cages without bedding. Faecal pellets were collected every 15 min, and the number produced was recorded as a measure of gut motility and function. The initial weight of the collected pellets was recorded for each mouse, then the pellets were dried at 60 °C overnight. The reduction in faecal weight post-drying (%) indicated faecal water content, an indirect measure of gastrointestinal water absorption as previously published [58].

#### Gut transit time assay

At 10 weeks old, mice were administered 150 μL of a filtered and autoclaved 6 % (w/v) dilution of non-absorbable carmine red dye (#C1022, Sigma-Aldrich) in 0.5 % methylcellulose (#M0512, Sigma-Aldrich), via oral gavage, according to a published protocol [71, 72]. Following dye administration in the morning, mice were single housed with *ad libitum* access to food and water to determine the latency to excretion of the first red-dyed faecal pellet as a measure of gut transit time. The experiment was ended after a maximum time of 9 h elapsed after carmine red administration.

#### Gut permeability assay

At 13 weeks of age, a randomly selected subset of mice from cohort 3 were fasted overnight, then administered 150 μL of 4 kDa fluorescein isothiocyanate-dextran (FITC-dextran; #FD4, Sigma-Aldrich) dissolved in PBS to a concentration of 100 mg/mL, via oral gavage. 4 h after administration of FITC-dextran, mice were terminally anaesthetised before blood collection via cardiac puncture. Plasma concentration (mg/mL) of FITC-dextran of individual samples was assessed using serial dilutions of FITC-dextran standards, with fluorescence of standards and plasma samples measured using a PHERAstar spectrophotofluorometer (BMG LABTECH, Ortenberg, Germany) as a measure of gut permeability [72, 73]. Plasma obtained from mice not fasted or administered FITC-dextran on each experimental day was used in assays to account for background fluorescence of plasma.

#### Calprotectin ELISA

Faecal calprotectin was assessed as a non-invasive and clinically relevant measure of gastrointestinal inflammation in a subset of cohort 3, using the Mouse S100A8/S100A9 Heterodimer DuoSet ELISA kit (#DY8596-05, R&D Systems, MN, USA). Faecal samples were suspended at a concentration of 100 mg/mL in a 0.1 % Tween-20 PBS solution on ice, and vortexed for up to 20 min until fully homogenised. Homogenate was centrifuged at 12000 RPM at 4 °C for 10 min and supernatant collected for use in ELISA, and stored at -20 °C until further processing. ELISA was performed according to manufacturer’s directions, and a four parameter logistic regression analysis was fitted to determine calprotectin concentrations (pg/mL) of faecal samples. Two technical replicates were included per sample, and samples were removed from the analysis based on coefficient of variation (CV, %) between these duplicates.

### Full-length 16S rRNA amplicon analysis

#### Faeces collection

Faecal pellets were collected from cohort 3 mice for 16S sequencing at 12 weeks of age. Mice were placed into individual cages without bedding, and provided with food and water, for one hour. Faecal pellets uncontaminated with urine were collected using sterile syringe needles. 0-4 pellets were collected per mouse, flash frozen on dry ice and stored at -80 °C until further processing of n = 8 randomly selected samples per genotype and sex groups.

#### Full-length 16S rRNA amplicon sequencing and bioinformatic analysis

Faecal samples were transferred to the Centre for Pathogen Genomics Innovation Hub, Department of Microbiology and Immunology, University of Melbourne, for downstream processing and sequencing. Full length (V1 - V9) 16S amplicons were generated using Oxford Nanopore Technologies’ standard 16S barcoding kit (SQK-16S114.24, Oxford Nanopore Technologies, Oxford, UK). Reads were filtered to only retain those longer than 1400 bp and shorter than 1700 bp to remove any non-16S sequences. Species-level taxonomy was assigned to the resulting reads by *Emu* [74]. One sample (male *+/+*) was removed prior to bioinformatic analysis due to low read numbers. Species abundance data was filtered to retain only features appearing in at least two samples. For alpha diversity analyses, Simpson dominance indices and Shannon entropy values were extracted from species abundance data using *Qiime2* [75] (version 2024.10), and statistically analysed for group differences using R [76] (version 4.2.0) by fitting a generalised linear model. For beta diversity analysis, species data were filtered to include those that have > 0.1 % relative abundance and was robust centred-log ratio transformed to calculate robust Aitchison distance for beta diversity analysis using the *vegan* R library [77, 78]. Omnibus significance was assessed using PERMANOVA and pairwise comparisons were performed to compare groups using *vegan* [77] and *RVAideMemoire* [79] libraries, respectively. For differential abundance analysis, stratified by sex due to observed sex differences in microbial composition, genotype effects were assessed using *MaAslin2* [80] and the *p*-values were adjusted using the Benjamini-Hochberg method to obtain *q*-values.

For functional prediction of microbiome changes, *PICRUSt2* [81] (Phylogenetic Investigation of Communities by Reconstruction of Unobserved States) was employed after processing full-length reads using *NaMeco* [82]. Reads were clustered into operational taxonomic units (OTUs), and representative reads for each cluster were polished. The resulting count table was used as input for *PICRUSt2*. For beta diversity analysis of pathway data, predicted MetaCyc pathways [83] were filtered to include those with relative abundance > 0.1 %, then robust centred-log ratio transformed to calculate Aitchison distance using *vegan*, with omnibus significance assessed using PERMANOVA and pairwise comparisons between groups performed using *vegan* and *RVAideMemoire* R libraries, as previously for compositional beta diversity analysis. Differential abundance testing was carried out stratified by sex using *Maaslin2*, using the Benjamini-Hochberg method to correct for multiple testing. Further pairwise comparisons were performed using the Wilcoxon rank-sum test.

For integrative analysis of 16S data, brain anatomical and gut functional data, the *DIABLO* [84] (Data Integration Analysis for Biomarker Discovery using Latent Variable Approaches for Omics Studies) method from the *mixOmics* [85] toolkit was applied to investigate their combined ability to discriminate between genotypes. This approach uses multiblock sparse partial least squares discriminant analysis (sPLS-DA) within an N-integration framework. A data subset was selected to maximise sample size and variable inclusion; representativeness of the subset was confirmed by comparing subset and full‑cohort summary statistics for all included variables (brain weight, body weight at 13 weeks, gut transit time, Bristol stool score, gut permeability). Resulting female *+/-* sample sizes were too low for inclusion in subsequent analysis (n = 2), therefore only males were included in the final *DIABLO* analysis.

### Tissue collection procedures

Where plasma was not required, mice were culled via cervical dislocation only. Where plasma was collected, mice were anaesthetised with sodium pentobarbitone (80 mg/kg in NaCl 0.9 %, intraperitoneal injection). Upon cessation of the pedal withdrawal reflex, blood was collected from the heart into EDTA-coated reagent tubes (#450531, Greiner Bio-One GmbH, Kremsmünster, Austria). Whole blood was centrifuged at 4 °C and 1000 G for 10 min to obtain plasma for gut permeability assays.

For all mice from cohorts 1 and 2, and a subset of mice from cohort 3, brains were extracted from the skull, olfactory bulbs and brain stem removed, and the whole brain was weighed using precision scales (0.1 mg accuracy) before microdissection of regions of interest. The prefrontal cortex, hippocampus and striatum were identified, dissected and weighed immediately before flash-freezing on dry ice and storage at -80 °C until further processing. Caecum and colon were dissected and lengths measured using a ruler. The caecum was additionally weighed using precision scales.

### Statistics and figures

**Fig. 2:**
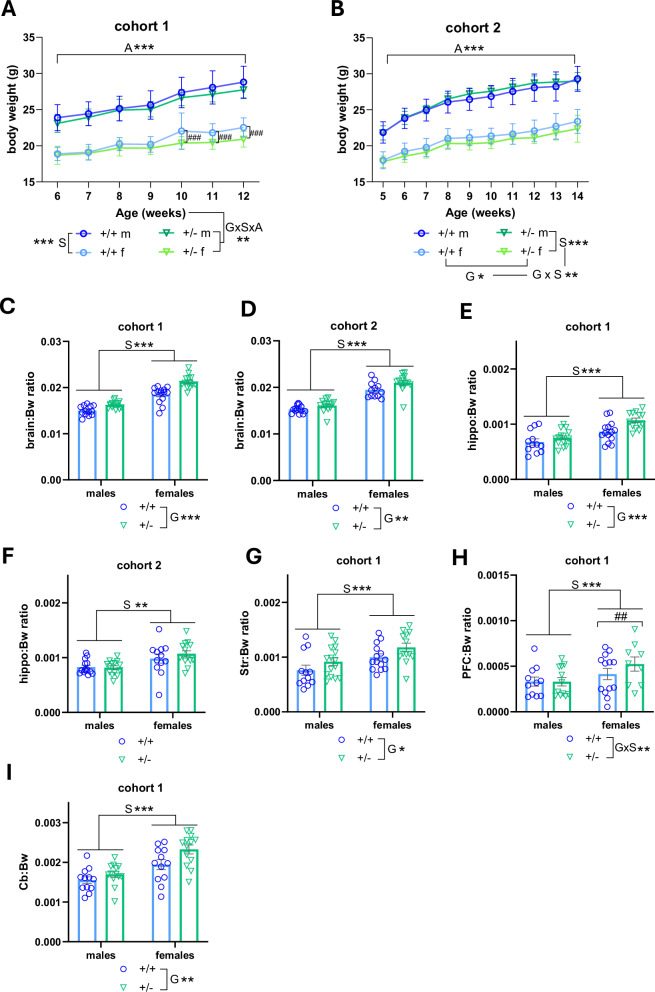
Body weight and brain anatomical measures in *Nf1***+**/- mice. In (**A**) cohort 1, body weight was significantly altered only in female *Nf1 +/-* mice at the age of 10, 11 and 12 weeks (Genotype x Sex x Age: *p* = 0.0025**; Sex x Age p = 0.0053** not shown on graph). In (**B**) cohort 2, no significant effects of Nf1 deficiency on body weight were observed according to post hoc pairwise comparisons despite significant main effects of Genotype, Sex, and interactions between Genotype, Sex, and Age (Sex x Age p < 0.0001*** not shown on graph). In both (**C**) cohort 1 and (**D**) cohort 2, *Nf1 +/-* mice showed an increased brain weight to body weight ratio (**C**, genotype: p < 0.001***; **D**, genotype: p = 0.0022**), while females similarly showed a significantly higher brain weight to bodyweight ratio (**C**, sex: p < 0.001***; D, sex: p < 0.001***) compared to their male counterparts. In cohort 1 (**E**), but not cohort 2 (**F**), *Nf1 +/-* mice showed an increased hippocampus to body weight ratio (**E**, Genotype: p = 0.0008***). In cohort 1, we also measured the weights of striata, prefrontal cortices and cerebella. *Nf1 +/-* mice of both sexes showed significantly increased ratios of (**G**) striatum weight to body weight (G, genotype: p = 0.0108*), while (**H**) prefrontal cortex weight to bodyweight ratio was altered specifically in females (H, Sex: p < 0.001; Genotype x Sex: p = 0.0091**; simple effects, Tukey-adjusted; f | *+/-* vs. *+/-* p = 0.0082**). (**I**) Cerebellum weight to bodyweight was also lower in *Nf1 +/-* animals (I, Genotype: p = 0.0043**). Mean values are shown with SEM error bars (**A, B**), or individual values are shown with mean and SEM error bars (**C-I**). * denotes p < 0.05, ** denotes p < 0.01 and *** denotes p < 0.001 for main effects and interactions; ### denotes p < 0.001 for pairwise comparisons using Tukey’s method. G: main effect of genotype; S: main effect of sex; A: main effect of age; G x S: interaction effect Genotype x Sex; G x S x A: interaction effect Genotype x Sex x Age; Bw: bodyweight; hippo: hippocampus; str: striatum; PFC: prefrontal cortex; Cb: cerebellum.

**Fig. 3:**
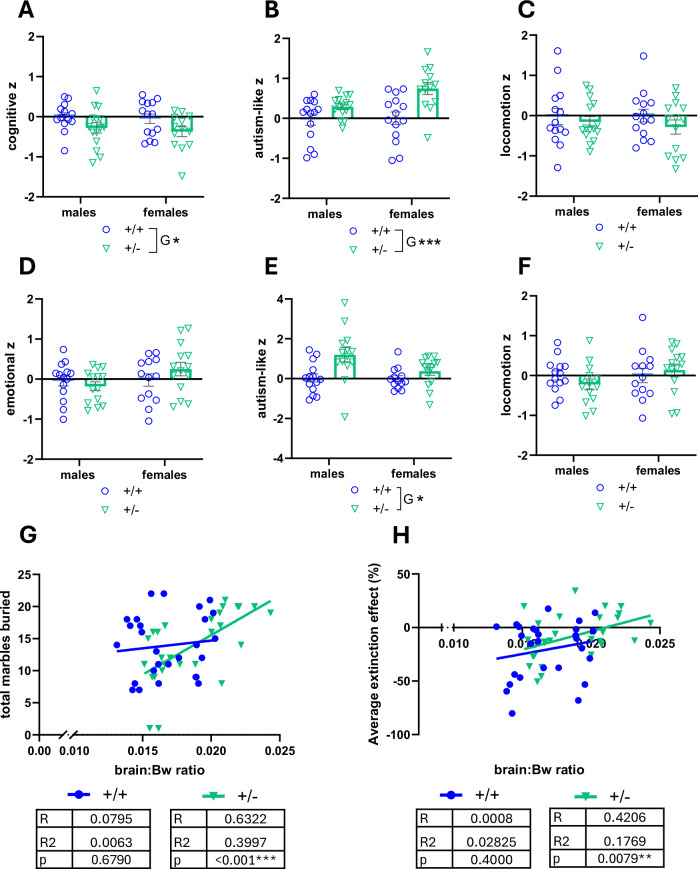
Integrative analysis of behaviour and brain-behavioural correlations in *Nf1***+**/- mice. In cohort 1 (**A–C**), when combining measures from all cognitive tests and corrected whole brain weight values, *Nf1 +/-* mice showed (**A**) a significantly reduced combined cognitive z-score (Genotype, p = 0.0207*). Across (**B**) autism-like parameters, which included PPI and marble burying results, and corrected whole brain weight values, *Nf1 +/-* mice showed significantly increased combined z-scores (**B**, Genotype, *p* < 0.001***), but showed no differences across (**C**) locomotion test z-scores. In cohort 2 (**D–F**), across (**D**) anxiety- and depression-like tests, mice showed no significant differences, suggesting unaltered emotional behaviour in *+/-* mice. In contrast, across (**E**) Autism-relevant measures such as the SIT and relative brain weight, *Nf1 +/-* mice again showed significantly increased combined z-scores (E, Genotype, p = 0.0173*). Similarly as for cohort 1, *+/-* mice showed no differences across (**F**) locomotion test z-scores in cohort 2. Simple linear regression analysis revealed that brain to body weight ratios significantly predicted (**G**) stereotypic behaviour in the marble burying test (*p* = 0.0079**), and (**H**) fear extinction effect in terms of difference in freezing between time blocks 9 and 1 of the test (*p* < 0.001***), suggesting a potential mechanistic link between these behavioural outcomes and the brain enlargement seen in *Nf1 +/-* mice. Individual values are shown with mean and SEM error bars. * denotes *p* < 0.05 and *** denotes *p* < 0.001. G: main effect of genotype.

**Fig. 4:**
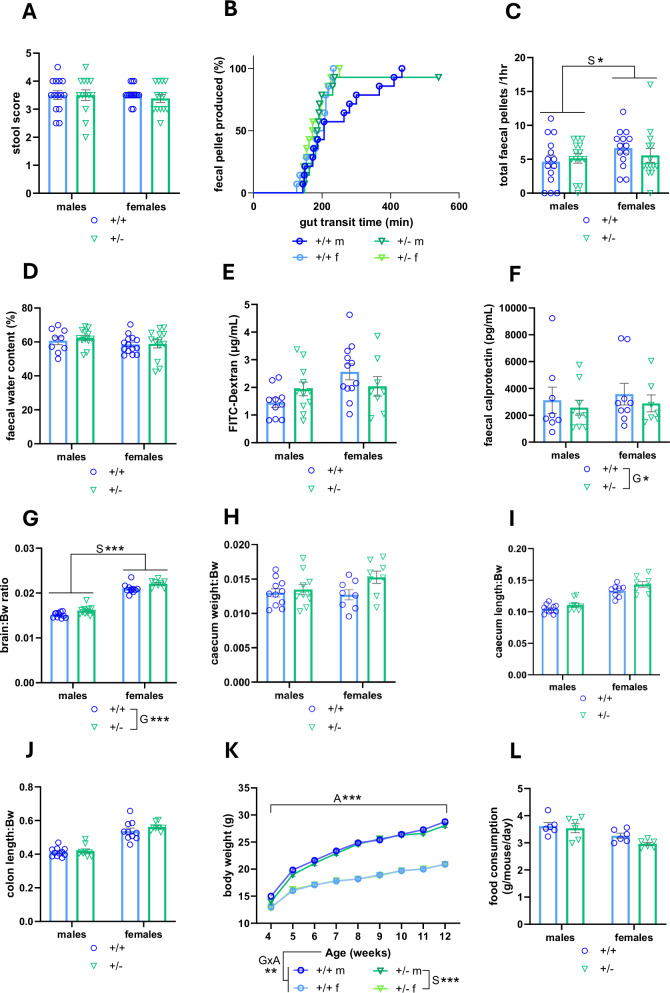
Gastrointestinal functional, physiological and anatomical measures in *Nf1***+**/- mice. Gastrointestinal parameters were evaluated by (**A**) faecal pellet output over an hour, demonstrating an increased rate of faecal pellet passage in females compared to males (A, Sex: p = 0.0420*), and **B** gut transit time, (**C**) Bristol stool score and (**D**) faecal water content which displayed no significant differences between *NF1 +/-* and *+/+* animals. Gut inflammation was evaluated by (**E**) faecal calprotectin concentration where no significant difference was observed. Gut permeability was evaluated by (**F**) FITC-dextran concentration in plasma 4 h after oral gavaging of a non-absorbable bolus of FITC-dextran, which showed no significant differences between Nf1-deficient mice and controls. An (**G**) increased brain weight to body weight ratio was demonstrated in *Nf1 +/-* mice compared to their *+/+* counterparts (G, Genotype, *p* < 0.001***; Sex: *p* < 0.001***). **(H)** Caecum weight to body weight ratio, (**I**) caecum length to body weight ratio and (**J**) colon length to body weight ratio showed no significant differences between experimental groups, suggesting neurofibromin deficiency does not impact gross indicators of GI tract anatomy or caecal function. The (**K**) body weight of *Nf1 +/-* in this cohort did not differ from *+/+* controls at any timepoint, contrary to previous cohorts (K, Sex, *p* < 0.001***; Age: *p* < 0.001***; GxA p = 0.0051**; pairwise comparisons n.s.). **L** Average food consumption by male and female *+/-* mice (measured per cage over a week) was similar to their control counterparts. Individual values are shown with mean and SEM error bars (**A, C-J, L**), or mean values are shown with SEM error bars (**K**). * denotes *p* < 0.05, ** denotes *p* < 0.01 and *** denotes *p* < 0.001 for significant main effects and interactions. G: main effect of genotype; S: main effect of sex; A: main effect of age; GxA: interaction effect Genotype x Age; Bw: body weight.

**Fig. 5:**
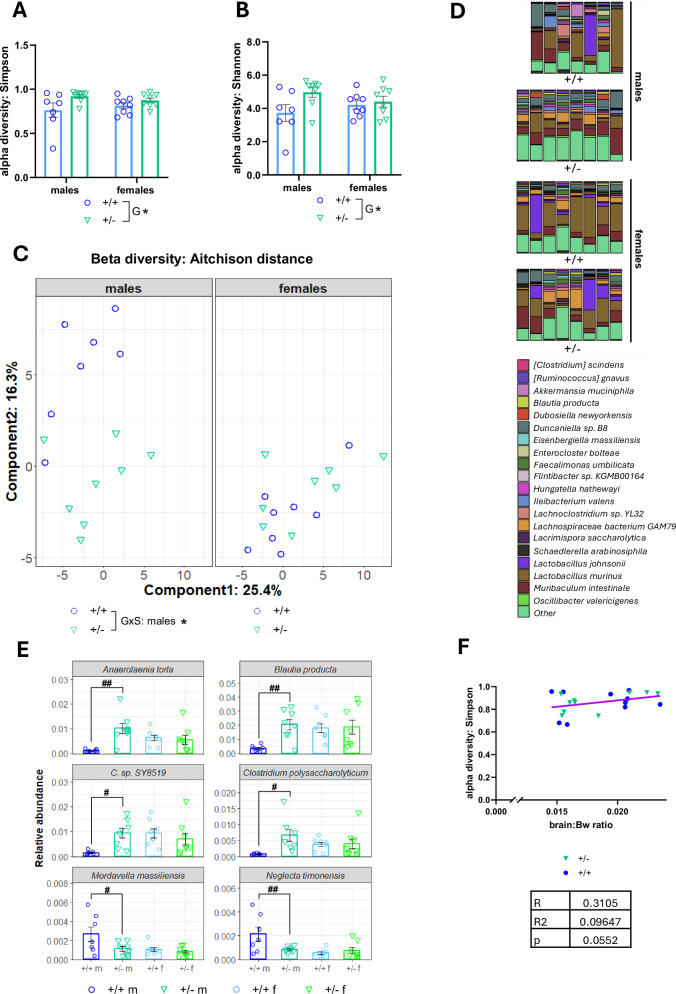
Compositional analysis of faecal microbiota in *Nf1***+**/- mice. Significantly increased alpha diversity was observed in the gut microbiota composition of *Nf1 +/-* mice compared to their wild-type counterparts in both (**A**) Simpson and (**B**) Shannon indices (A, Genotype, p = 0.0309*; B, Genotype, p = 0.0191*). Increased beta diversity, as determined by analysing the (**C**) Aitchison distance across two principal components. Male *Nf1 +/-* mice and male *+/+* mice showed a statistically significant difference in this measure, indicating altered overall microbial community diversity between males lacking neurofibromin and control males (C, Genotype x Sex, p = 0.0240*; pairwise comparison males only - *+/+* vs. *+/-* p = 0.0120*). (**D**) Relative species abundance per sample in male and female, *+/-* and *+/+* mice is shown, with the top 20 most abundant species across all samples represented by different colours (see accompanying legend). Species-level differential abundance analysis showed no species differences in *+/-* females, however male *Nf1 +/-* faecal samples had (**E**) six species significantly over- or underrepresented compared to controls (*Anaerotaenia torta* | Genotype q = 0.0012##; *Clostridium sp. SY8519* | q = 0.028#; *[Clostridium] polysaccharolyticum* | q = 0.0352#; *Blautia producta* | q = 0.0093##; *Neglecta timonensis* | q = 0.0081##; *Mordavella massiliensis* | q = 0.0122#). Differentially abundant species between male *Nf1 +/-* and *Nf1* *+/+* mice are shown for all groups in six panels. (**F**) Interestingly, brain to body weight ratios and Simpson’s indices were marginally positively correlated (p = 0.0552) in a subset of animals where brain weight data was collected. Individual values are shown with mean and SEM error bars (**A, B**) with box-plots (**E**), individual values are shown (**C**), or relative abundance of species (**D**). * denotes *p* < 0.05 result of linear mixed-model analysis; # denotes q < 0.05; ## denotes q < 0.01 corrected p values of differential abundance analysis using Maaslin2. G: main effect of genotype; GxS: interaction effect of genotype by sex.

**Fig. 6:**
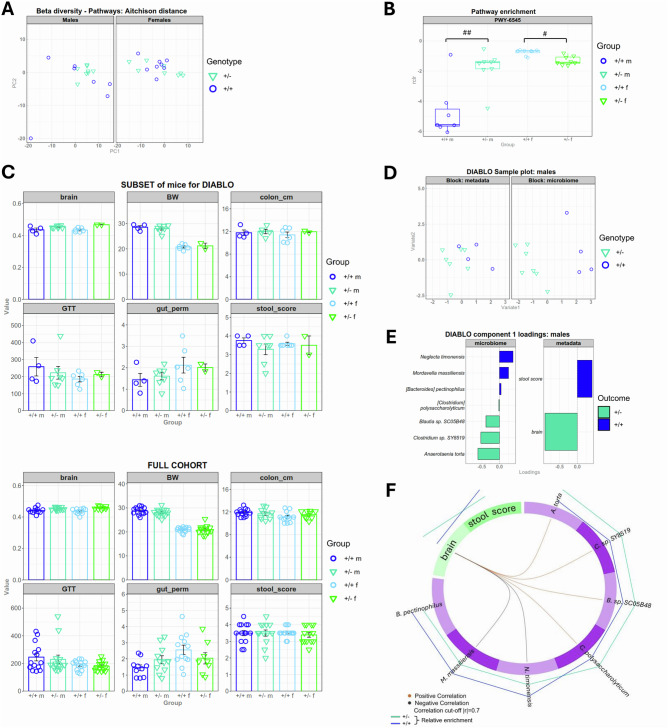
Predicted functional capacity and multi-omic signature of faecal microbiota in *Nf1***+**/- mice. Functional pathway predictions of compositional 16S data revealed (**A**) no overall genotype differences in beta diversity using robust Aitchison distances, but a significant sex difference was observed (Sex: p = 0.023*). **(B**) A single pathway, PWY‑6545 (pyrimidine deoxyribonucleotides de novo biosynthesis III) emerged as differentially abundant in *Nf1 +/-* mice in both males and female groups (*Maaslin2* analysis: m | Genotype: q = 0.0091**; f | Genotype: q = 0.0323*). Further analysis (Wilcoxon rank-sum pairwise comparison: m | *+/+* vs. *+/-* p = 0.042*; f | *+/+* vs. *+/-* p = 0.015*; *+/+* | m vs. f p = 0.007**; *+/-* m vs. f p > 0.05) revealed that directionality of these changes was in opposing directions. Supervised integration analysis was performed after selection of a (**C**) representative data subset, leading to the inclusion of brain weight, body weight (at 13 weeks), gut transit time, stool score, and gut permeability parameters, in addition to 16S data. While low sample counts in females precluded further analysis, in males, (**D**) a host-microbial signature consisting of seven microbial species, brain weight, and stool score, was identified as capable of discriminating between genotypes. (**E**) Brain weight and stool score were found to inversely correlate with each other, while individually correlating with specific microbial species’ levels, as shown in loading plot for latent component 1 (blue: positively associated with +/+ outcome, green: positively associated with +/- outcome). (**F**) Circos plot shows strong positive and negative correlations (Pearson’s |r| > 0.7) of microbial expression levels with brain weight (orange lines: positive correlation |r| > 0.7; dark grey lines: negative association |r| > 0.7). Radial lines show direction of association of each microbial species and metadata measurement (brain weight, Bristol stool score) with genotype outcomes according to the DIABLO model. # denotes Wilcoxon rank-sum pairwise comparison results p < 0.05; ## denotes p < 0.01 (only between-genotype comparisons shown on graph). Individual values (**A, D**), or individual values with boxplots representing means and interquartile ranges (**B**), individual values with mean and SEM error bars (**C**), or bars showing loading values for selected features (**E**) are shown. rclr: robust centred-log transformed ratio, DIABLO: Data Integration Analysis for Biomarker Discovery using Latent Variable Approaches for Omics Studies.

Graphs for Figs. 1–5 were plotted using GraphPad Prism (version 10) as dot plots of raw values with means, and with error bars representing the standard error of the mean. Graphs for Fig. 6 were prepared in GraphPad Prism or R [76] (version 4.2.0) and RStudio (version 2024.04.0, Build 735) using *ggplot2* [86]. Data were analysed in R with the use of packages *lme4* [87]*, lmerTest* [88], *glmmTMB* [89]*, car* [90], for the generation of linear models, generalised linear models, and linear mixed models, and *emmeans* [91] for pairwise comparisons in the event of significant interactions of main factors.

The Shapiro-Wilk test was used to determine normal distribution of datasets and suitability of model where appropriate, then analysed with a linear mixed-effects model if normal, or a generalised linear mixed-effects model if non-normal. ‘Genotype’ and ‘Sex’ were the fixed factors for all measures, while ‘Age’, ‘Day’, ‘Time’, ‘Intensity’ or ‘Block’ were added for repeated-measures analyses. Main effects and interaction effects were evaluated with a significance level alpha (α) = 0.05. *Post hoc* pairwise comparisons were performed using Tukey’s method when significant interaction effects were observed. For linear regression analyses, the R package *robustbase* [92] was used. For time-to-event datasets, where some individuals failed to reach a pre-defined outcome, survival analysis using Cox regression (proportional hazards) was implemented (via the *coxme* [93] R package).

A preliminary analysis revealed that both *cage* and *litter* significantly contributed to the observed variation in a number of behavioural tests to varying degrees, therefore both were included as random factors where applicable. We also included *dissector* as a random factor to control for variability in brain dissection technique across experimenters, or *day* when experiments were performed across several days to account for batch effects. For 16S analyses, the majority of samples came from different cages and litters, and thus accounted for these effects in the experimental design, therefore these factors were not included in the statistical analysis.

Sample sizes were determined based on our previous extensive experience in behavioural neuroscience research (including [49, 51, 53, 64, 67]). To detect effects of moderate to large sizes, considered appropriate in preclinical research, we aimed to include n = 10-15 mice per sex and genotype group for behavioural and anatomical analyses, whereas for all molecular analyses (ELISA, gut permeability, faecal 16S sequencing), at least n = 7 mice per group were included per sex and genotype group.

Supplementary table 2 includes a full list of sample sizes, statistical tests including applied random factors and covariates for each analysis, results of normality testing, and exact *p*- or *q*-values. No outliers were excluded, as datasets were considered well-powered and therefore should not be susceptible to significant skewing by extreme values. Other reasons for excluding animals from statistical analyses are listed in Supplementary table 2 for each analysis.

## Results

### Nf1-deficient mice display no significant alterations in stereotypic, anxiety- and depression-like behaviours, but show increased feeding behaviour after fasting

*Nf1 +/-* and *+/+* controls, a widely established mouse model used in NF1 research [22], were subjected to an extensive battery of standard behavioural tests in two separate cohorts (Fig. 1A), evaluating behaviours across multiple domains. *Nf1 +/-* mice of both sexes did not show any significant changes in anxiety-like behaviour across the large open field test (Supplementary figure 1A), the light-dark box test (Supplementary figure 1B) or the elevated plus maze (Supplementary figure 1C), in which they spent similar proportions of time in the aversive zones of the respective arenas. In the novelty-suppressed feeding test, *+/-* mice and *+/+* controls showed similar feeding behaviour in a novel and anxiogenic environment (Supplementary figure 1D), and lost equivalent amounts of body weight during the 24-hour fast prior to the test (Supplementary figure 1E; main effect - Sex: *p* = 0.0459), though they consumed significantly more food during the 5 min immediately after the test, indicating higher levels of hunger in the *+/-* mice after a fast (Supplementary figure 1F; main effect — Genotype: *p* = 0.0222), and precluding any interpretation from the latency test (Supplementary figure 1D) relating to depression- or anxiety-like behaviour. Regarding depression-like behaviour, *+/-* mice did not show alterations in saccharin preference (Supplementary figure 1G) in the saccharin preference test over two days, nor in behavioural despair in the Porsolt swim test (Supplementary figure 1H, I). *Nf1 +/-* mice buried similar numbers of marbles during the marble burying test as their control counterparts, suggesting neurofibromin deficiency did not impact stereotypic behaviour (Supplementary figure 1J). Considering the frequent diagnosis of ADHD in individuals with NF1, we also evaluated locomotor activity. Total distances travelled were unchanged across tests in *+/-* mice, suggesting this mouse model does not show overt hypo- or hyperactivity (Supplementary figure 2A-D). Importantly, this meant that locomotion was not a confounding factor in the interpretation of other behavioural tests.

### Neurofibromin-deficient mice show reduced sociability but no overt changes in learning and memory, or motor control

Short-term spatial memory in the Y maze (Fig. 1B) and object memory in the novel object recognition test (NOR; Fig. 1C) were not significantly different between *+/-* and *+/+* controls, as indicated by the respective indices showing similar levels of preferential exploration of a novel arm (Y maze) and novel object (NOR) across experimental groups. In another test of spatial memory, the Morris water maze, both neurofibromin-deficient mice and controls successfully acquired the location of a hidden platform and improved their recall performance over 5 training days, though there was a tendency for an overall reduced performance in *+/-* mice (Fig. 1D; main effects – Genotype: *p* = 0.084; Time: *p* < 0.001). On the probe day of the test, there was no difference in the time taken to reach the former platform location between *+/+* and *+/-* groups (Fig. 1E), indicating similar levels of spatial learning recall. Sensorimotor gating was evaluated using the prepulse inhibition test (Fig. 1F-H). Overall, there were no differences between genotype groups in average startle amplitude (F; main effect – Sex: *p* = 0.0052), nor in the startle response inhibition by a prepulse stimulus when delivered either 30 ms (G; main effect - Intensity: *p* < 0.001) or 100 ms (H; main effect – Intensity: *p* < 0.001) before the primary auditory stimulus. No effect of neurofibromin deficiency was found in associative learning between a tone and a mild foot-shock in the fear conditioning test, although *Nf1 +/-* showed higher levels of freezing at baseline, which was therefore implemented as a covariate (Fig. 1I; main effects – Block: *p* < 0.001; Block 0 freezing – Genotype: *p* = 0.0446). Fear extinction training was performed 24 h after fear conditioning. Here, the tone stimulus is given in absence of a foot shock in blocks 1-9, which did not reveal any differences between *+/+* and *+/-* mice (Fig. 1J; main effect – Block: *p* < 0.001). Thus, surprisingly, *Nf1 +/-* mice showed no overt changes in the primary outcomes of several standard paradigms evaluating learning and memory.

During the first phase of the social interaction test, *+/-* mice were less sociable (Fig. 1K; main effect – Genotype: *p* = 0.0361; interaction – Genotype x Sex: *p* = 0.0651), as they spent relatively less time than *+/+* controls in the compartment containing an unknown mouse compared to the empty compartment. This effect seemed to be driven predominantly by the males, as indicated by the trend for a Genotype x Sex interaction. In contrast, social recognition memory was not impacted by neurofibromin deficiency in either sex, as all groups preferentially interacted with the novel mouse rather than a familiar one (Fig. 1L). In a test of motor coordination, the Rotarod, *+/-* mice performed similarly to *+/+* controls across three trials, indicating neurofibromin deficiency does not impact motor control (Supplementary figure 2E).

### Nf1 deficiency in mice leads to body weight and brain anatomical changes

Mice from cohorts 1 (Fig. 2A) and 2 (Fig. 2B) were weighed weekly. In cohort 1, significant sex- and age-dependent differences were observed, where female *+/-* showed a significantly reduced weight from weeks 10-12 compared to female *+/+*, with marginal significance at weeks 8 and 9 of age (cohort 1, A; main effects – Age; *p* < 0.001; Sex: *p* < 0.001; interactions — Sex x Age: *p* = 0.0053; Genotype x Sex x Age: *p* = 0.0025; relevant simple effects, Tukey-adjusted: 8 wks | f | *+/+* vs. *+/- p* = 0.0649; 9 wks | f | *+/+* vs. *+/- p* = 0.0749; 10 wks | f | *+/+* vs. *+/- p* < 0.001; 11 wks | f | *+/+* vs. *+/- p* < 0.001; 12 wks | f | *+/+* vs. *+/- p* < 0.001). Interestingly, cohort 2 mice deficient in neurofibromin did not show similar differences in body weight despite significant main effects of Genotype and several interactions suggesting subtle differences (Fig. 2B; main effects – Genotype: *p* = 0.0195; Age: *p* < 0.001; Sex: *p* < 0.001; interactions – Genotype x Sex: *p* = 0.0070; Sex x Age: *p* < 0.001; relevant simple effects, Tukey-adjusted: all n.s.).

As a gross measure evaluating structural anatomical differences, the weight of freshly extracted brains and individual dissected brain areas were recorded and compared between groups. When correcting for body weight, *+/-* mice from both cohort 1 (Fig. 2C; main effects – Genotype: *p* < 0.001; Sex: *p* < 0.001) and cohort 2 (Fig. 2D; main effects – Genotype: *p* = 0.0022; Sex: *p* < 0.001) had significantly higher relative brain masses compared to *+/+* controls. In cohort 1, increased relative weight was also observed in the hippocampus (Fig. 2E; main effects – Genotype: *p* = 0.0008; Sex: *p* < 0.0001), and the striatum (Fig. 2G; main effects – Genotype: *p* = 0.0108; Sex: *p* < 0.001). In the prefrontal cortex, only *+/-* females showed an increase in brain region:body weight ratio (Fig. 2H; main effect — Sex: *p* < 0.001; interaction — Genotype x Sex: *p* = 0.0091; relevant simple effects, Tukey-adjusted; f | *+/+* vs. *+/- p* = 0.0082). The cerebellum of cohort 1 mice was also of lower relative weight (Fig. 2I; main effects – Genotype: *p* = 0.0043; Sex: *p* < 0.001) in neurofibromin-deficient mice. In contrast, *+/-* mice from cohort 2 had similar corrected hippocampal weights as the *+/+* control mice (Fig. 2F). Other brain tissues were not collected from cohort 2.

In cohort 2, additional gut macroscopic measurements of the caecum (weight, length) and colon (length) were taken upon dissection to examine the impact of neurofibromin deficiency on the digestive tract. While the analysis of raw measurements suggested a significant effect of genotype on these parameters (data not shown), body weight-corrected caecum and colon measurements did not differ between *+/+* and *+/-* groups (Supplementary figure 3), suggesting that any observed differences were related to overall subtle size differences between mice.

### Combined z-scores evaluating behavioural domains reveal cognitive deficiency and consistent autism-like phenotype in *Nf1 +/-* mice

While the rodent behavioural tests carried out in cohort 1 and 2 were designed to detect significant effects of moderate to large size, a more comprehensive approach was deemed appropriate in the *Nf1 +/-* mouse model to detect more subtle changes in the different behavioural domains. To this end, we employed a quantitative approach using combined z-scores [69] to summarise the phenotypes of individual mice across several different, but related, test parameters and measurements (Supplementary table 1). In cohort 1, a combined cognitive z-score was calculated for each mouse, revealing that *Nf1 +/-* mice exhibited significantly poorer performance across all cognitive tests as indicated by a reduced cognitive z-score (Fig. 3A; main effect – Genotype: *p* = 0.0207). Of the tests performed in cohort 1, prepulse inhibition and marble burying, as well as brain weight measurements, were considered relevant for the autism-like symptom cluster seen in many individuals with NF1. The combined autism-like z-score resulting from these measures showed a striking increase in *Nf1 +/-* mice compared to *+/+* controls, an effect more pronounced in female *+/-* mice (Fig. 3B; main effect – Genotype: *p* < 0.001; Sex: *p* = 0.0532; interaction — Genotype x Sex *p* = 0.0594). In contrast, a locomotion z-score combining distances travelled across all relevant tests did not reveal a hyper- or hypoactive phenotype in neurofibromin-deficient mice of cohort 1 (Fig. 3C).

*Nf1 +/-* mice from cohort 2 showed similar combined emotional z-scores as *+/+* controls (Fig. 3D), suggesting an absence of behavioural differences across tests relevant to depression and anxiety. We chose to omit the scores of relevant tests involving appetitive behaviour (novelty-suppressed feeding test and saccharin-preference test) in this calculation due to the observed alterations in feeding behaviour in *Nf1 +/-* (see Supplementary figure 1F). The autism-like z-score in cohort 2 was derived from sociability and relative brain weight measures for each animal, and was significantly higher in the *Nf1 +/-* group, though here the difference was more pronounced in males (Fig. 3E; main effect – Genotype: *p* = 0.0098; interaction — Genotype x Sex: *p* = 0.0582). As previously, no differences in the combined locomotion z-score were observed in cohort 2 (Fig. 3F).

To determine whether relative brain size was associated with any of the evaluated behavioural outcomes, we performed simple linear regression analysis. This analysis revealed that in *+/-* mice, but not *+/+* mice, the brain:body weight ratio was strongly or moderately predictive of performance in the marble burying test (Fig. 3G; for *+/-* R = 0.6238; R2 = 0.3821; *p* < 0.001) and the fear extinction paradigm, respectively (Fig. 3H; for *+/-* R = 0.4206; R2 = 0.1769; *p* = 0.0079). These findings provide the first evidence that there may be a significant link between the pathophysiological processes leading to increased brain size and those underlying behavioural alterations in *Nf1 +/-* mice.

### Neurofibromin deficiency does not impact digestive tract function but produces a significant decrease in gut inflammatory marker levels

In a separate cohort of *+/+* and *+/-* mice, several measures of gut function, physiology and anatomy were evaluated (Fig. 4). Frequency of faecal pellet production (Fig. 4A) and latency to excrete a faecal pellet containing a red dye, administered orally to measure gut transit time, were unchanged in *+/-* mice (Fig. 4B). Consistency of faecal pellets was scored according to the Bristol stool scale [94] (Fig. 4C) and water content was determined (Fig. 4D), both of which were also unchanged by neurofibromin deficiency. Faecal calprotectin levels were measured as an indicator of gut inflammation, and these were found to be significantly reduced in *Nf1 +/-* mice compared to control mice across sexes (Fig. 4E; main effects – Genotype: *p* = 0.0200; Sex: *p* = 0.0830). On the other hand, gut permeability in neurofibromin-deficient mice was not altered, as demonstrated by similar levels of plasma FITC-Dextran 4 h after oral administration (Fig. 4F; main effect – Sex: *p* = 0.0052; interaction — Genotype x Sex: *p* = 0.0476; relevant simple effects, Tukey-adjusted: all n.s.).

As in previous cohorts, there was a highly significant increase in brain weight relative to body weight in *Nf1 +/-* mice compared to *+/+* controls (Fig. 4G: main effects – Genotype: *p* < 0.001; Sex: *p* < 0.001). However, gut anatomical measurements, including bodyweight-adjusted caecum weight (Fig. 4H), caecum length (Fig. 4I) and colon length (Fig. 4J) were not significantly different between groups. In this cohort, body weight over time of neurofibromin-deficient mice was similar to control mice of the same sex and age (Fig. 4K; main effects – Sex: *p* < 0.001; Age: *p* < 0.001; interaction — Genotype x Age: *p* = 0.0051; relevant simple effects, Tukey-adjusted: all n.s.). Food intake per cage, measured over one week and averaged per day and per mouse, also did not differ between male or female *+/-* mice and *+/+* control counterparts, further supporting that there were no overt metabolic differences in this cohort (Fig. 4L).

### Neurofibromin-deficient mice show gut microbiota composition changes reminiscent of autism spectrum disorder

Faeces from male and female Nf1 *+/+* and *+/-* mice were collected at 12 weeks of age to perform a comparative analysis of gut microbiota composition using full-length 16S rRNA sequencing. Alpha diversity, measuring microbiota community richness within individual samples, was found to be overall significantly higher in neurofibromin-deficient mice across sexes in both Simpson’s dominance (Fig. 5A; main effect – Genotype: *p* = 0.0309), and Shannon entropy indices (Fig. 5B; main effect – Genotype: *p* = 0.0191). However, in both measures this effect seems driven primarily by alterations in the *+/-* male group. Beta diversity, which is a measure of diversity between different communities (groups of individuals) was evaluated using Aitchison distance. In males, neurofibromin deficiency was associated with a significant difference in microbiota composition compared to the control group, as indicated by the clear separation between *+/-* and *+/+* groups (Fig. 5C, left panel; PERMANOVA main effect – Sex: *p* = 0.0010; interaction — Genotype x Sex: *p* = 0.0240; pairwise PERMANOVA between groups: m | *+/+* vs. *+/- p* = 0.0120). In females, however, no differences in beta diversity were observed between the *+/+* and *+/-* groups (Fig. 5C, right panel).

Microbiota composition was further evaluated by examining the abundance of individual bacterial species in the gut of *Nf1 +/+* and *+/-* mice of both sexes (Fig. 5D). There were no differences in relative abundance of specific species in females, however statistical analysis revealed that in the faecal samples of male *Nf1 +/-*, four species of bacteria were more abundant than in *+/+* controls, while two species were significantly less abundant in *+/-* than in *+/+* mice (Fig. 5E; adjusted *q*-values for male comparisons - *Anaerotaenia torta*: *q* = 0.0012; *Clostridium sp. SY8519*: *q* = 0.0280; *Clostridium polysaccharolyticum*: *q* = 0.0350; *Blautia producta*: *q* = 0.0093; *Neglecta timonensis*: *q* = 0.0080; *Mordavella massiliensis*: *q* = 0.0122). Interestingly, in a subset of animals where both brain weight data and 16S alpha diversity data were available (*+/+* males n = 4; *+/+* females n = 6; *+/-* males n = 7; *+/-* females n = 3), linear regression analysis showed that Simpson’s alpha diversity index had a tendency to predict relative brain weight across *+/-* and *+/+* groups, with higher brain-to-body weight ratios seen in mice with higher alpha diversity values (Fig. 5F; R = 0.3105; R2 = 0.0965; *p* = 0.0552). While the correlation was weak to moderate, this finding underscores a potential relationship between the observed brain and gut microbiome phenotype resulting from neurofibromin deficiency in NF1.

### Microbiota composition changes in *+/-* mice suggest limited functional impacts, while supervised integration of gut function, brain anatomy and 16S data reveals host-microbe signatures of neurofibromin deficiency in males

To delve further into the potential consequences of microbiota composition changes in *Nf1 +/-* mice, reported above, we next performed functional pathway prediction from the generated taxonomic profiles using *PICRUSt2* [81]. Pathway beta diversity analysis showed a significant difference between sexes, but no effects for Genotype or Genotype x Sex interaction (Fig. 6A, PERMANOVA main effect – Sex: *p* = 0.0230). Pairwise PERMANOVA testing between individual groups revealed no significant differences after correcting for multiple testing. Despite the lack of a global genotype effect on pathway composition, differential abundance testing (stratified by sex as for compositional beta diversity analysis) using *Maaslin2* revealed a single pathway that showed differing levels of enrichment according to genotype (Fig. 6B; main effect – Genotype: *q* = 0.0091). Interestingly, the change was in opposing directions: The ‘pyrimidine deoxyribonucleotides de novo biosynthesis III’ (PWY-6545) pathway was significantly upregulated in male *Nf1 +/-* (Fig. 6B; pairwise Wilcoxon test between groups: m | *+/+* vs. *+/- p* = 0.042), while it was significantly downregulated in *+/-* females compared to *+/+* (Fig. 6B; pairwise Wilcoxon test between groups: f | *+/+* vs. *+/- p* = 0.015).

While the impact on predicted functional pathways thus appeared relatively limited, the subtle correlation between alpha diversity and brain anatomical measurements, described above, suggests a potential mechanistic link between the gut microbial alterations in *Nf1 +/-* mice, and other key phenotypes investigated here. We therefore sought to determine whether any aspects of the phenotype observed in the cohort of *+/-* and *+/+* mice that provided faecal samples for 16S microbiota analysis were associated with specific microbiome alterations. To this end, we employed *DIABLO* supervised integration analysis [84, 85], allowing the selection of features that show maximal covariance, combined with maximal discriminatory power between *+/+* and *+/-* genotypes. Parameters selected for the analysis (in addition to the 16S datasets) were brain weight, body weight (at 13 weeks), gut transit time, Bristol stool score, and gut permeability. This maximised the final sample sizes for *DIABLO* analysis, while including a wide array of observations and ensuring that the subset of samples included in *DIABLO* were representative of the full cohort across selected measures (Fig. 6C). Colon length was initially included, however the 16S cohort was found to have less representative values in this parameter (6 C). In addition, this preliminary analysis reduced the female *+/-* subgroup to n = 2 which was insufficient for further analysis, and was therefore only performed in males. Tuning of the model led to the identification of an optimal signature of seven microbial species and two phenotypic measurements (brain weight and stool score) that were simultaneously associated with each other, while also able to discriminate between *+/+* and *+/-* genotypes (Fig. 6D), representing a discriminatory signature between *+/+* and *+/-* males that combines host and microbial features. Brain weight and stool softness score each correlated with abundance levels of particular microbial species, while negatively correlating with each other (Fig. 6E). Of particular interest for our investigation into the microbiota-gut-brain axis in *Nf1 +/-*, four microbial species were strongly positively correlated with brain weight: *Clostridium sp. SY8519* (R = 0.8549281), *Anaerotaenia torta* (R = 0.8368198), *Clostridium polysaccharolyticum* (R = 0.8023390) and *Blautia sp. SC05B48* (R = 0.7662035); while three showed a negative correlation with brain weight: *Mordavella massiliensis* (R = -0.7610691), *Neglecta timonensis* (R = -0.7355181) and *Bacteroides pectinophilus* (R = -0.6916692), as shown in Fig. 6F.

## Discussion

In the current study, we employed rigorous and integrative experimental and statistical approaches to substantially expand on the currently published literature in the *Nf1 +/-* mouse model and, crucially, to identify an alteration in gut microbiota composition in a heterozygous NF1 mouse model for the first time. We show that *Nf1 +/-* mice display a behavioural phenotype that replicates the cognitive deficits and autistic traits, as well as a brain enlargement phenotype, as seen in individuals with NF1. Importantly, we show that the expression of these cognitive-behavioural traits in individual mice is concordant with brain mass differences, supporting a potential mechanistic association. Finally, in male *Nf1 +/-* mice, we identify brain weight, Bristol stool score, and seven microbial species as a host-microbial signature that covaries and robustly discriminates *+/-* from *+/+* mice, strongly suggesting that the gut microbiota may be implicated in genotype-associated differences in brain phenotype.

We first set out to broadly characterise the behaviour of both male and female *Nf1 +/-* mice which carry a single mutated copy of the *Nf1* gene. As males and females are equally affected by NF1, the absence of a female *+/-* group in many published studies to date is regrettable, although a recent study has started to address this major research gap [95]. We performed a range of tests investigating anxiety- and depression-like behaviour, none of which revealed a significantly altered phenotype in *Nf1 +/-* mice. This replicates previously published findings in males using this model [32], despite the fact that anxiety and depression occur at higher rates in individuals with NF1 than in the general population. Nevertheless, we also found that *Nf1 +/-* mice had a higher hunger drive after fasting in the novelty-suppressed feeding test, which is consistent with previous findings that *Nf1 +/-* mice have lower serum leptin and higher serum ghrelin levels; although they generally do not consume more food at baseline [96], which is also reflected in our findings. This phenotype directly interferes with performance on the novelty-suppressed feeding test and may also affect results from the saccharin-preference test, a behavioural paradigm used to measure anhedonia. As a result, reliable measurement of depression-like behaviour in these mice is limited to the Porsolt swim test, which showed no differences between groups. Indeed, the combined z-score incorporating measures from each test of anxiety and depression-like behaviours showed no genotype differences, supporting the notion that these symptom complexes do not develop in the *Nf1 +/-* mouse model.

Regarding learning and memory, despite well-powered cohorts and rigorous methodology, we were unable to replicate several previously reported deficits in the literature. Most notably, spatial learning in the Morris water maze appeared largely unaffected in both male and female *+/-* mice despite several studies having previously reported this phenotype [21, 23, 24]. Methodological differences may explain this, since unusually large sample sizes [21, 24] and/or differences in the number of training and probe trials [23] would allow detection of smaller effects and may vary the difficulty of the task, respectively. It has also been suggested that the C57BL/6 background may reduce the emergence of the phenotype relative to hybrid backgrounds [31]. The experimental design used in the present study has been validated extensively by our group, however the effect size commonly seen in *Nf1 +/-* mice may be too small to detect with standard cohort sizes. Our integrative approach using combined z-scores [68, 69], on the other hand, was able to detect the subtle reduction in learning and memory performance shown by *Nf1 +/-* across the different paradigms. While the effect remains small, the unified approach employed here has distinct advantages, as a statistically significant difference between groups indicates that individuals showed concordant cognitive deficits across different tests.

In this context, one of the most striking phenotypes we observed was an increase in a combined z-score relating to different autism-relevant traits in each of the three examined cohorts. In each cohort, we first considered which data sets were relevant to autism, had previously been used in similar autism-like z-score calculations [69], or were closely related to autism symptoms. This led to the calculation of two different ‘autism-like z-scores’; each incorporating those available datasets for each individual mouse of a given cohort. Remarkably, while several of the individual effects were small (sociability index reduction) or absent (marble burying, prepulse inhibition), the combination of distinct variables together with individual relative brain weights revealed that the *Nf1 +/-* in each cohort showed consistently higher scores in these combined parameters. As a novel result highly relevant to NF1, the body-weight-corrected brain weight measure, found to be significantly higher in *Nf1 +/-* mice across all cohorts, was included in all of the autism-like z-scores. Previous research into brain volume in *Nf1 +/-* mice had already revealed subregion-specific enlargement of the striatum and prefrontal cortex regions using morphometric MRI measurements [25]. This result was partially replicated in cohort 1, where we found the striatum (which comprises the caudate putamen) to be relatively heavier in *+/-* mice across sexes, as well as the prefrontal cortex being heavier in female *+/-* mice. As we did not measure these brain areas in all cohorts, it is difficult to determine whether these changes are robust effects of neurofibromin deficiency. Hippocampal weight differences were not consistent across cohorts, suggesting that subregion-specific changes were less robust than whole-brain effects, or that effect sizes were too small to detect with the sample sizes employed here. However, the aforementioned study did not include whole brain MRI measurements and comparisons, despite the number of reports in the human NF1 literature consistently showing increased volumes across grey matter and white matter [33–39, 97–100]. Neurofibromin plays an important role in neural progenitor proliferation and differentiation, and has been shown to be involved in neural specification [101, 102], which likely underlies the striking phenotype observed. It is important to note that while several of the *Nf1 +/-* behavioural phenotypes reported here and in the literature are known to be relatively subtle, and potentially moderated by the genetic background [26, 31], the brain mass phenotype was apparent across all examined cohorts. It was not dependent on a change in body weight, as this was only observed in females from cohort 1 at specific timepoints – an effect we suspect may be related to stress, as the most stressful behavioural tests (Morris water maze, prepulse inhibition and fear conditioning) coincided with the weight measurement differences. We additionally found relevant behavioural measures, average fear extinction and marble-burying behaviour, were significantly correlated with the brain enlargement in *Nf1 +/-* mice, but not in *+/+* mice. We did not find that social interaction or recognition, nor spatial-learning performance, were significantly correlated with any brain alterations, which had previously been shown in this mouse model using MRI-based morphometric measurements in brain subregions [25]. However, the findings from our integrative behavioural evaluation using z-scores suggest that the brain enlargement in individual *+/-* tends to be concordant with autism-relevant behaviours. Taken together, these results provide strong evidence for a link between the robust brain phenotype we observed, and the behavioural changes caused by neurofibromin deficiency. Importantly, studies into the relationship between brain size and neuropsychological symptoms performed several decades ago predominantly found similar associations [33, 35, 38, 99, 103–105], although the exact nature of this relationship is still not understood. Collectively, our findings provide convincing evidence that the *Nf1 +/-* mouse model will be very useful in the study of the potential causal involvement of brain enlargement (and other neural abnormalities) in NF1 cognitive and psychiatric symptoms.

Despite the expanding literature supporting gut microbiome alterations as a contributing factor to many brain disorders, including autism, this association has never been experimentally investigated in NF1 cognitive and psychiatric symptom complexes, nor in the heterozygous mouse model of NF1. Almost a decade ago, Beales published a theoretical paper positing the involvement of ‘biome depletion’ in the pathogenesis of NF1 [106]. Although microbiota were included in this broad definition, the theory was mainly based on the proposed involvement of macrobiotic helminths, a type of parasitic worm linked to reduced inflammation, particularly in the formation of plexiform neurofibromas [106]. The gut microbiota was also suggested by Gutmann and colleagues as a possible risk factor contributing to tumorigenesis in NF1, based on a collection of findings in mouse models of NF1 [107]. In the present study, we show for the first time that heterozygous *Nf1 +/-* mice, which do not develop tumours until an advanced age, have a significantly altered gut microbiota composition. We therefore propose that changes in gut microbiota composition may play a role in the pathophysiological mechanisms of NF1 leading to the brain and behavioural phenotype seen in this disorder. The reported tendency for the brain weight to correlate with alpha diversity, a measure of microbiota community richness, first hinted at this possibility. Integrative analysis of the 16S rRNA sequencing dataset with gut parameters and brain weight measurements was then carried out, and further strengthened this proposed mechanistic association in males. While we were unable to carry out the *DIABLO* analysis in females due to missing data, the absence of a clear Genotype effect of neurofibromin deficiency on gut microbiota composition (or indeed most gut parameters) in females, with the significant alpha diversity effects appearing to be driven mainly by males, means that we would not expect to see substantial results in the integrative analyses in females, however its absence is a limitation of our study. In males, the analysis revealed a correlation between brain weight, Bristol stool score, and the abundance of seven microbial species (*Clostridium sp. SY8519, Anaerotaenia torta*, *Clostridium polysaccharolyticum, Blautia sp*. SC05B48, *Mordavella massiliensis*, *Neglecta timonensis* and *Bacteroides pectinophilus;* several of which unsurprisingly overlap with differentially abundant species in males), and together these features were able to discriminate between *+/+* and *+/-* individuals. With five species strongly correlating with brain weight, and three species strongly correlated with stool score, the results thus indicate that the genotype-driven gut microbial variations are linked to specific host phenotypes. Interestingly, stool score was not significantly different between *Nf1 +/+* and *+/-* mice in the main analysis, further highlighting the power of integrative approaches in uncovering more subtle phenotypic differences. Importantly, the fact that the most strongly covarying and discriminatory host-microbial signature was found to include brain weight (at the model tuning phase of this analysis) provides compelling evidence in support of a link between brain enlargement and gut microbiota changes in NF1, at least in males. Interestingly, several studies examining brain structure and microbiota composition have noted a significant association between specific gut microbial parameters or taxa, and brain structure in humans, including in healthy [108, 109], obese [110], and depressed [111] individuals. In mice, modulation of the gut microbiota has been reported to influence brain structure [112, 113]. While the mechanisms and specific involvements of particular species or taxa are still difficult to pinpoint at this stage, these studies collectively support the notion that gut microbiota changes can influence brain structure, both in physiological and in pathophysiological circumstances. Intriguingly, while the results of our differential analysis of predicted functional pathways only revealed a single differentially enriched pathway, the ‘pyrimidine deoxyribonucleotides de novo biosynthesis III’ (PWY-6545), the directionality of this change was opposite in males and females, with pathway enrichment compared to controls found in male *+/-* mice while depletion compared to controls was observed in females. The significance of these changes for the observed gut microbiota changes, and the overall *Nf1 +/-* phenotype, cannot be unequivocally determined without further knowledge about the precise function of altered microbial species (in males), and further experimental validation of this functional effect (e.g. via shotgun metagenomics and metabolomic analyses). Pyrimidine deoxyribonucleotides (dNTPs) are required for DNA synthesis, thus changes in their availability may impact cell proliferation. This effect is likely locally restricted, mainly impacting microbial metabolism and proliferation, or in the case of epithelium-associated species, that of host cells in physical proximity [114, 115]. However, it is interesting to note that recent studies in an African population have found an association between this pathway’s predicted function in the individual’s microbiome, and early life growth (including height, weight, and growth velocity) [116], as well as its predicted activity in the maternal microbiome during gestation and birth weight [117], tying in with the idea that dNTPs may be salvaged by host cells in periods of high demand [114]. However, it is still entirely unclear whether this association extends to brain development specifically, and/or to other human populations. Future longitudinal studies will also need to examine whether the gut microbiota of *Nf1 +/-* in early life, including during key periods of brain development, are similarly functionally changed as in the current study. While microbiota composition has surprisingly never been examined in individuals with NF1, there has been a very recent study investigating a potential role for the gut microbiota in tumour formation in the optic glioma (OPG) mouse model of NF1 [118]. In this model, *Nf1* is completely knocked out in many parts of the CNS via Cre recombinase-mediated recombination in GFAP-expressing cells, i.e. in all cells, including neurons and astrocytes, derived from GFAP-expressing neural stem cells [119]. This preclinical model is therefore useful in the study of brain tumour formation and tumour microenvironments in the disorder. Genetically, *Nf1 +/-* mice employed in this study may have superior construct validity, although they do not show loss of heterozygosity to the same extent as individuals with NF1, including in the brain—which may account for the subtlety of the behavioural phenotype seen here. In the OPG model, Chatterjee *et al*. provide evidence that optic glioma formation is dependent on their gut microbial population, as germ-free mice carrying the relevant mutation did not develop optic nerve growths to the same extent as conventionally-reared OPG mice [118]. Furthermore, long-term antibiotic treatment partially inhibited optic nerve proliferation in this model, and faecal matter transplant from normally reared OPG mice to germ-free and antibiotic-treated OPG mice partially restored the OPG phenotype, suggesting that microbial populations present in the OPG gut flora may be driving tumour development in the brain [118]. This study thus demonstrates that the gut microbiota may contribute to tumour formation, although a number of questions remain to be answered, including whether OPG mice have a different gut microbial population compared to control animals at baseline. Overall, the findings indicate that brain neurofibromin deficiency and gut microbiota changes may interact to influence the tumour microenvironment and support the gut microbiota as a potential target for treatment of brain tumours in NF1.

Taken together, our findings in *Nf1 +/-* mice and those from the OPG model represent powerful evidence that the gut microbiota plays a crucial role in the brain of individuals with NF1—potentially influencing both brain development and tumour progression in the context of neurofibromin deficiency.

The proposed role for gut microbial alterations in the cognitive and autism-related symptomatology seen in individuals with NF1 is further supported by the increasing numbers of studies reporting a significant association between autism and changes in the gut microbiota [120]. In fact, several studies have reported an increase in alpha diversity and/or beta diversity in subjects with autism [121–123] similar to the increase shown in our *Nf1 +/-* male mice by 16S rRNA sequencing analysis, although there have also been inconsistent results regarding these diversity measures [122, 124, 125]. While it has been suggested that these differences may be primarily driven by altered dietary preferences [125], we are able to exclude this possibility in mice, which receive identical diets across all experimental groups. Studies into specific alterations in microbiota composition have started to uncover a number of differentially enriched orders, families and genera of bacteria [121–123, 126, 127]. In particular, a recent systematic review examined the published literature and found that both clinical and preclinical studies of autism and gut microbiota populations have shown increased abundance of microbiota belonging to the genera *Clostridium*, and to the family *Lachnospiraceae*, both of which are represented in the species that we found to be enriched in the faecal microbiota populations of *Nf1 +/-* males [120] (*Clostridium sp*. *SY8519*, *Clostridium polysaccharolyticum*; and *Anaerotaenia torta*, *Blautia producta*, respectively). On the other hand, another finding in clinical and preclinical faecal samples has been a reduction in the species belonging to the *Blautia* genus [120, 128, 129], while we found that *Blautia producta* was significantly more abundant in *Nf1 +/-* males than controls. However, it should be noted that *Blautia producta* belongs to the *Blautia* genus, as well as the *Lachnospiraceae* family, therefore the previously published findings appear to partially contradict each other. The increase in *Lachnospiraceae* appears to be the more robust finding, reported by numerous preclinical and clinical studies, and reflects what we saw in the *Nf1 +/-* mice [126, 128, 130–134]. Interestingly, some of these associations are thought to be important determinants of gastrointestinal symptoms in individuals with autism, which are relatively common [135]. NF1, on the other hand, is less commonly associated with GI dysfunction, and where gut symptoms are present they are most often linked to tumour formation. While we did not observe any obvious deficits in the examined parameters relating to gut physiology and function in *Nf1 +/-* mice, it is still unclear whether this accurately reflects the situation in NF1. We did observe a reduction in the inflammatory marker calprotectin, which is commonly used in the diagnosis of inflammatory bowel syndrome. Calprotectin has crucial functions in immune defence [136], therefore concentrations below normal control levels could potentially indicate lower-than-normal immune responsivity in the gut of *Nf1 +/-* mice, however further assays will be needed to confirm this interpretation. Overall, while it remains unclear whether the microbiota and gut functional changes we observed have overall positive or negative health effects, the resemblance to preclinical models of autism in terms of gut microbiota composition is remarkable, and should be further explored, including as a potential treatment avenue.

An important limitation of our study design is that we were unable to directly link the changes in gut microbiome composition observed in *+/-* mice to the alterations in behaviour, due to faecal collections being performed in a different cohort of mice, which were subjected predominantly to tests of gut function. Furthermore, animals in the behavioural cohorts were co-housed across genotypes, which may have led to sharing of microbiota between groups. This may have diluted the cognitive-behavioural phenotypes, especially if microbiota are partially driving phenotypes, and future studies should always keep genotypes housed separately to avoid confounds. Nevertheless, we were able to present the first evidence of a link between the behaviours shown by *Nf1 +/-* mice and increased brain size, and via a separate analysis in males, found that brain weight was strongly associated with gut microbial expression and stool softness. These findings allow us to infer that there may be an important mechanistic link between the gut microbiota, brain structure, and behaviour, in male *Nf1 +/-*, which will require targeted follow-up studies to establish causal relationships.

As mentioned above, we specifically included male and female mice to be able to determine the influence of sex as a biological variable in brain, behavioural and gut microbiome phenotypes in the *Nf1 +/-* mouse model. Studies of individuals with NF1 show similar prevalence of the disorder between males and females, however there appear to be slight differences in symptomatology, including progression of certain tumours [137], self-reported quality of life and emotional symptoms [138], and autistic behaviours [48, 139], though the underlying mechanisms are unknown. Across behaviours, we found no significant sex effects, however it is interesting to note that the Genotype effect in the sociability index analysis was accompanied by a statistical trend for a Genotype x Sex interaction, and visual examination of the data confirms that the effect is mainly driven by a difference in male *+/-* animals. Interestingly, several studies [139, 140] (but not all [141]) have reported that social dysfunction is greater in male individuals with NF1, reflecting our result and suggesting a biological (rather than societal) basis for this particular sexual dimorphism. On the other hand, the gut microbiome alterations were mainly apparent in males, with beta diversity unchanged, and alpha diversity showing very small changes in the female group. Also, while a functional pathway was predicted to be impacted in females, this change was in the opposite direction and smaller in amplitude than in the males. Overall, our data indicates that the gut microbiota changes due to neurofibromin deficiency in females are limited, compared to males, however this does not exclude the possibility that sex differences in the gut microbiome may be driving differences in symptomatology. Conversely, it is possible that the microbiome contributes differently to particular symptoms even if they are equally observed in *+/-* males and females, such as with the brain enlargement observed across both sexes (and linked to the microbiota phenotype in males by integrative analysis). As many pertinent questions remain unanswered, we strongly argue for the inclusion of females in all future studies of the microbiota-gut-brain axis in NF1.

In conclusion, we were able to demonstrate that the *Nf1 +/-* mouse model is highly suitable for the investigation of NF1 symptom clusters related to autism and cognition, and provide novel evidence supporting its use in the study of brain volume increases consistently seen in individuals with NF1. The significant changes in gut microbiota composition, especially in male *Nf1 +/-* mice, and its link to brain structural changes need to be urgently extended to clinical NF1 populations, as it raises the possibility of new treatments via therapies targeting the gut microbiome. Such novel therapeutic interventions might be particularly promising with respect to the poorly treated cognitive and psychiatric symptoms.

## Supplementary information

Supplementary Figures and Tables

## Data Availability

Datasets generated by this study will be made publicly available in online data repositories upon publication (16S sequencing data: NCBI Sequence Read Archives (SRA) under BioProject PRJNA1441776; other datasets – Figshare: 10.6084/m9.figshare.31839700).

## Code availability

Code used for 16S and integrative statistical analyses is available on github: https://github.com/gkong1/Reisinger_et_al_2026_Mol_Psych.
