# Supplementary material for: Gut microbiome alterations are sex-dependently associated with brain abnormalities in a mouse model of Neurofibromatosis type I
Source: Mol Psychiatry. 2026 Apr 27;31(9):5184–203. doi: 10.1038/s41380-026-03609-0 (PMC13441884; doi:10.1038/s41380-026-03609-0)
Supplement: Supplementary file 1 [file 41380_2026_3609_MOESM1_ESM.docx]

Reisinger SN, *et al.*
**Gut microbiome alterations are sex-dependently associated with brain abnormalities in a heterozygous mouse model of Neurofibromatosis type I**

**SUPPLEMENTARY FIGURES AND TABLES**

Full details of statistical tests, results and sample sizes can be found in Supplementary table 2.

**Supplementary figure 1.** Male and female +/+ and +/- mice were subjected to behavioural testing for evaluation of emotional behaviours. Anxiety-like behaviour was evaluated in the (A) large open field test, the (B) light-dark box test, and the (C) elevated plus maze, neither of which revealed any significant differences in behaviour in +/- mice in terms of time spent (%) in the aversive zones of the respective tests. (D) In the novelty-suppressed feeding test (NSFT), depression-related anxiety was evaluated by measuring levels of hyponeophagia in mice, which was not different between groups. Accompanying control measurements for the NSFT revealed that Nf1 +/- mice (E) lost similar amounts of weight after a 24-hour fast as +/+ controls (Sex: p= 0.0459*), but (F) consumed significantly more food immediately after the NSFT trial (Genotype: p= 0.0222*) suggesting higher levels of hunger drive and confounding the interpretation of this test relating to emotional behaviour. Depression-like behaviour was unchanged by neurofibromin deficiency (G-I). (G) In the saccharin preference test, anhedonia was not different between genotypes. +/- mice of both sexes also showed similar levels of behavioural despair in the Porsolt swim test as the control group, since they showed similar levels of floating (H) and did not differ in the timing of their first immobility bout (I). Stereotypic behaviour, as determined by the number of marbles buried in the (J) marble burying test, was not different between +/+ and +/- groups of either sex. Individual values are shown with mean and SEM error bars. * denotes p<0.05. G: main effect of genotype; S: main effect of Sex.

**Supplementary figure 2.** Total distances travelled during locomotion tracking-based behavioural tests and motor coordination did not differ between Nf1 +/+ and +/- mice of either sex. +/+ and +/- mice travelled similar distances in (A) the large open field test, (B) the light-dark box test, (C) the test trial of the novel object, and (D) the social interaction test, indicating that mice lacking one copy of the neurofibromin gene did not show overt hyper- or hypoactivity. (E) Nf1 +/- showed similar performance as +/+ controls in the Rotarod test, indicating comparable motor coordination. Individual values are shown with mean and SEM error bars.

**Supplementary figure 3.** Macroscopic measurements of gut anatomy in cohort 2 Nf1 +/+ and +/- mice did not differ between genotypes when corrected for body weight. (A) Relative caecum weight was similar between +/+ and +/- groups, as was the (B) relative length of the caecum. (C) Colon length, relative to body weight, was also similar between +/+ and +/- groups. Significance for sex effects is not shown. Individual values are shown with mean and SEM error bars.

| **Combined z-score** | **Cohort** | **Figure** | **Experiment** | **Parameters included** |
| --- | --- | --- | --- | --- |
| Cognitive  z-score | 1 | 4A | Morris water maze (MWM), fear conditioning (FC), fear extinction (FE), Y maze (YM), prepulse inhibition (PPI) | MWM: latency to platform (training day 5), latency to platform location (probe day); FCC: freezing difference block 6 vs. 1; FCE: freezing difference block 9 vs. 1; YM: novelty preference index; PPI: average PPI at 30 ms prepulse, average PPI at 100 ms prepulse. |
| autism-like  z-score 1 | 1 | 4B | Marble burying (MB), prepulse inhibition (PPI), brain weight | MB: number of marbles buried; PPI: average PPI at 30 ms prepulse, average PPI at 100 ms prepulse; relative brain weight |
| autism-like  z-score 2 | 2 | 4E | Social interaction test (SIT), brain weight | SIT: sociability index, relative brain weight |
| Locomotion z-score 1 | 1 | 4C | Morris water maze (MWM), Y maze (YM) | MWM: total distance training, total distance probe day; Y maze: total distance (trial 2) |
| Locomotion z-score 2 | 2 | 4F | Large open field (LOF), light-dark box test (LDB), elevated plus maze (EPM), Porsolt swim test (PST), social interaction test (SIT); novel object recognition test (NOR) | LOF, LDB, EPM, SIT, NOR: total distance; PST: immobility |
| Emotionality z-score | 2 | 4D | Large open field (LOF), light-dark box test (LDB), elevated plus maze (EPM), Porsolt swim test (PST), social interaction test (SIT) | LOF: time spent in centre; LDB: time spent in light zone; EPM: time spent in open arms; PST: immobility, latency to float; SIT: sociability index. |

**Supplementary table 1.** Combined z-scores were calculated for several behavioural and phenotypic domains for both cohorts of mice, encompassing a range of relevant measures from the same mice. This approach allows a more integrative analysis of the +/- phenotype compared to individual behavioural paradigms. Z-scores were first calculated for each mouse for individual test parameters (where several parameters from a single experiment were used), which were averaged for each mouse to generate a z-score per behavioural test or endophenotype. Test z-scores were then averaged for specific domains (cognition, autism-like traits, locomotion, emotionality) to generate a combined z-score per mouse. These were statistically analysed to evaluate differences between +/+ and +/- mice of both sexes. Included parameters and tests or phenotypes are listed in the table.

| **Experiment** | **Parameter** | **Figure** | **Cohort** | **Sample size** | **Exclusions** | **Shapiro-Wilk normality test** | **Main effects** | **Interactions** | **Multiple comparisons (where interaction p< 0.05)** | **Random factors or covariates** |
| --- | --- | --- | --- | --- | --- | --- | --- | --- | --- | --- |
| Y maze test | novelty preference index | 1B | 1 | +/+ males n= 13; +/+ females n= 14; +/- males n= 15; +/- females n= 13 | none | p= 0.9511 | Genotype p= 0.3112; Sex p= 0.9137 | Genotype x Sex p= 0.8571 | n/a | litter, cage |
| novel object recognition test | recognition index | 1C | 2 | +/+ males n= 14; +/+ females n= 10; +/- males n= 14; +/- females n= 13 | n= 4 removed: climbed onto objects | p= 0.7455 | Genotype p= 0.1810; Sex p= 0.2077 | Genotype x Sex p= 0.8126 | n/a | litter, cage |
| Morris water maze | latency to platform (s) over training days | 1D | 1 | +/+ males n= 13; +/+ females n= 14; +/- males n= 15; +/- females n= 13 | none | p= 0.0001*** | Genotype p= 0.0837; Sex p= 0.2729; Day p= 1.859e-07*** | Genotype x Sex p= 0.4396 | n/a | litter, cage |
| Morris water maze | latency to platform location (s) during probe trial | 1E | 1 | +/+ males n= 13; +/+ females n= 14; +/- males n= 15; +/- females n= 13 | none | p= 2.568e-06*** | Genotype p= 0.1270; Sex p= 0.3320 | Genotype x Sex p= 0.7750 | n/a | litter, cage |
| prepulse inhibition | average startle response (A.U) | 1F | 1 | +/+ males n= 13; +/+ females n= 13; +/- males n= 15; +/- females n= 13 | n= 1 removed: technical issue with PPI chamber | p= 0.0116* | Genotype p= 0.3742; Sex p= 0.0052** | Genotype x Sex p= 0.6977 | n/a | litter, cage |
| prepulse inhibition | prepulse inhibition (%), 30 ms | 1G | 1 | +/+ males n= 13; +/+ females n= 13; +/- males n= 15; +/- females n= 13 | n= 1 removed: technical issue with PPI chamber | p= 3.539e-06 *** | Genotype p= 0.7735; Sex p= 0.0519; Intensity p= 2e-16*** | Genotype x Sex p= 0.8706 | n/a | litter, cage |
| prepulse inhibition | prepulse inhibition (%), 100 ms | 1H | 1 | +/+ males n= 13; +/+ females n= 13; +/- males n= 15; +/- females n= 13 | n= 1 removed: technical issue with PPI chamber | p= 0.0022** | Genotype p= 0.4712; Sex p= 0.6757; Intensity p= 2e-16*** | Genotype x Sex p= 0.7257 | n/a | litter, cage |
| fear conditioning | freezing (%) | 1I | 1 | +/+ males n= 13; +/+ females n= 14; +/- males n= 15; +/- females n= 13 | none | p= 2.2e-16*** | Genotype p= 0.7212; Sex p= 0.5510; Block p= 2e-16***; Block 0 freezing p= 0.0446* | Genotype x Sex p= 0.7870; Genotype x Block p= 0.9734; Sex x Block p= 0.9835; Genotype x Sex x Block p= 0.9880 | n/a | litter, cage, Block 0 freezing (Genotype difference determined in preliminary analysis) |
| fear extinction | freezing (%) | 1J | 1 | +/+ males n= 13; +/+ females n= 14; +/- males n= 15; +/- females n= 13 | none | p= 5.874e-09*** | Genotype p= 0.7562; Sex p= 0.7910; Block p= 6.804e-05*** | Genotype x Sex p= 1138; Genotype x Block p= 0.6857; Sex x Block p= 0.2328; Genotype x Sex x Block p= 0.6698 | n/a | litter, cage |
| social interaction test | sociability index (trial 1) | 1K | 2 | +/+ males n= 14; +/+ females n= 13; +/- males n= 14; +/- females n= 14 | none | p= 9.023e-05*** | Genotype p= 0.0361*; Sex p= 0.1799 | Genotype x Sex p= 0.0651 | n/a | litter, cage |
| social interaction test | social recognition index (trial 2) | 1L | 2 | +/+ males n= 14; +/+ females n= 13; +/- males n= 14; +/- females n= 14 | none | p= 0.1877 | Genotype p= 0.9526; Sex p= 0.2223 | Genotype x Sex p= 0.8434 | n/a | litter, cage |
| body weight, cohort 1 | body weight (g) | 2A | 1 | +/+ males n= 13; +/+ females n= 14; +/- males n= 15; +/- females n= 13 | none | p = 7.44e-08*** | Genotype p= 0.1268; Sex p= 2.2e-16***; Age p= 2.2e-16 *** | Genotype x Sex p= 0.6602; Genotype x Age p= 0.6966; Sex x Age p= 0.0053**; Genotype x Sex x Age p= 0.0025** | (relevant only) | litter, cage |
|  |  |  |  |  |  |  |  |  | Males n.s. at all ages; females 6-7 wks old: n.s.; females 8 wks old: +/+ vs +/-: p= 0.0649; females 9 wks old: +/+ vs +/-: p= 0.0749; |  |
|  |  |  |  |  |  |  |  |  | females 10 wks old: +/+ vs +/-: p<0.0001***; females 11 wks old: +/+ vs +/-: p= 0.0002***; females 12 wks old: +/+ vs +/-: p<0.0001*** |  |
| body weight, cohort 2 | body weight (g) | 2B | 2 | +/+ males n= 14 ; +/+ females n= 13; +/- males n= 14; +/- females n= 14 | none | p= 8.372e-10*** | Genotype p= 0.0195*; Sex p= 2.2e-16***; Age p= 2.2e16 *** | Genotype x Sex p= 0.0070**; Genotype x Age p= 0.7122; Sex x Age p= 3.28e-11***; Genotype x Sex x Age p= 0.0997 | (relevant only) | litter, cage |
|  |  |  |  |  |  |  |  |  | Males +/+ vs +/-: p= 0.1212; females +/+ vs +/- p= 0.5262 |  |
| brain weight to body weight ratio, cohort 1 | brain weight to body weight ratio | 2C | 1 | +/+ males n= 13; +/+ females n= 14; +/- males n= 15; +/- females n= 12 | n= 1 removed: ‘dissector’ information missing | p= 0.0388* | Genotype p= 3.294e-05 *** ; Sex p= 2.2e-16 *** | Genotype x Sex p= 0.3719 | n/a | litter, cage, dissector |
| brain weight to body weight ratio, cohort 2 | brain weight to body weight ratio | 2D | 2 | +/+ males n= 13; +/+ females n= 12; +/- males n= 14; +/+ females n= 14 | n= 2 removed: brain weight information missing (1); body weight information missing (1) | p= 0.0193* | Genotype p= 0.0022** ; Sex p= 2e-16*** | Genotype x Sex p= 0.6453 | n/a | litter, cage, dissector |
| hippocampus to body weight ratio | hippocampus to body weight ratio | 2E | 1 | +/+ males n= 12; +/+ females n= 14; +/- males n= 15; +/- females n= 12 | n= 2 removed: ‘dissector’ information missing (1); dissection error (1) | p= 0.3532 | Genotype p= 0.0008***; Sex p= 8.915e-10*** | Genotype x Sex p= 0.0900 | n/a | litter, cage, dissector |
| hippocampus to body weight ratio | hippocampus to body weight ratio | 2F | 2 | +/+ males n= 14 ; +/+ females n= 12; +/- males n= 14; +/- females n= 14 | n= 1 removed: body weight information missing | p= 0.1747 | Genotype p= 0.2541; Sex p= 0.0048** | Genotype x Sex p= 0.1287 | n/a | litter, cage, dissector |
| striatum weight to body weight ratio | striatum weight to body weight ratio | 2G | 1 | +/+ males n= 12; +/+ females n= 14; +/- males n= 15; +/- females n= 12 | n= 2 removed: ‘dissector’ information missing (1); dissection error (1) | p= 0.2109 | Genotype p= 0.0108* ; Sex p= 2.577e-08 *** | Genotype x Sex p= 0.6101 | n/a | litter, cage, dissector |
| prefrontal cortex weight to body weight ratio | prefrontal cortex weight to body weight ratio | 2H | 1 | +/+ males n= 11; +/+ females n= 13; +/- males n= 12; +/- females n= 9 | n= 10 removed: ‘dissector’ information missing (1); dissection quality (9) | p= 0.0204* | Genotype p= 0.3870; Sex p= 5.562e-05 *** | Genotype x Sex p= 0.0091* | Males +/+ vs +/- p= 0.3184; females +/+ vs +/- p= 0.0082** | litter, cage, dissector |
| cerebellum weight to body weight ratio | cerebellum weight to body weight ratio | 2I | 1 | +/+ males n= 12; +/+ females n= 12; +/- males n= 13; +/- females n= 11 | n= 6 removed: ‘dissector’ information missing (1); dissection quality or weight missing (5) | p= 0.2937 | Genotype p= 0.0043** ; Sex p= 0.0001*** | Genotype x Sex p= 0.1776 | n/a | litter, cage, dissector |
| cognitive z-score | cognitive z-score | 3A | 1 | +/+ males n= 13; +/+ females n= 14; +/- males n= 15; +/- females n= 13 | none | p= 0.4094 | Genotype p= 0.0207* ; Sex p= 0.7751 | Genotype x Sex p= 0.9414 | n/a | litter, cage |
| autism-like z-score | autism-like z-score | 3B | 1 | +/+ males n= 13; +/+ females n= 14; +/- males n= 15; +/- females n= 13 | none | p= 0.7835 | Genotype p= 0.8026e-07***; Sex p= 0.0532 | Genotype x Sex p= 0.0594 | n/a | litter, cage |
| locomotion z-score | locomotion z-score | 3C | 1 | +/+ males n= 13; +/+ females n= 14; +/- males n= 15; +/- females n= 13 | none | p= 0.4404 | Genotype p= 0.1612 ; Sex p= 6281 | Genotype x Sex p= 0.5867 | n/a | litter, cage |
| emotionality z-score | emotionality z-score | 3D | 2 | +/+ males n= 14 ; +/+ females n= 13; +/- males n= 14; +/- females n= 14 | none | p= 0.2865 | Genotype p= 0.6267; Sex p= 0.1755 | Genotype x Sex p= 0.1138 | n/a | litter, cage |
| autism-like z-score | autism-like z-score | 3E | 2 | +/+ males n= 14 ; +/+ females n= 13; +/- males n= 14; +/- females n= 14 | none | p= 6.857e-10 | Genotype p= 0.0173*; Sex p= 0.4031 | Genotype x Sex p= 0.3292 | n/a | litter, cage |
| locomotion z-score | locomotion z-score | 3F | 2 | +/+ males n= 14 ; +/+ females n= 13; +/- males n= 14; +/- females n= 14 | none | p= 0.7645 | Genotype p= 0.8159; Sex p= 0.2617 | Genotype x Sex p= 0.1962 | n/a | litter, cage |
| brain vs behaviour correlation | Brain to body weight ratio vs. marble buried | 3G | 1 | +/+ males n= 9; +/+ females n= 14; +/- males n= 13; +/- females n= 13 | n= 6 removed: climbed out of arena | n/a | +/+: R=0.0795 \| R2=0.0066 \| p= 0.6790; +/-: R=0.6322; R2=0.3997; p= 5.75e-05*** | n/a | n/a | n/a |
| brain vs behaviour correlation | Brain to body weight ratio vs. average fear extinction | 3H | 1 | +/+ males n= 13; +/+ females n= 14; +/- males n= 15; +/- females n= 13 | none | n/a | +/+: R=0.0282 \| R2=0.0008 \| p= 0.400; | n/a | n/a | n/a |
|  |  |  |  |  |  |  | +/-: R=0.4206; R2=0.1769; p= 0.0079** |  |  |  |
| faecal pellet output | total faecal pellets/1hr | 4A | 3 | +/+ males n= 14; +/+ females n= 14; +/- males n= 14; +/- females n= 14 | none | p= 0.0579 | Genotype p= 0.5824; Sex p= 0.0743 | Genotype x Sex p= 0.2799 | n/a | litter, cage |
| gut transit time | time to red faecal pellet produced | 4B | 3 | +/+ males n= 14; +/+ females n= 14; +/- males n= 14; +/- females n= 14 | none | n/a | Genotype p= 0.962 ; Sex p= 0.424 | Genotype x Sex p= 0.604 | n/a | litter, cage |
| Bristol stool score | Bristol stool score | 4C | 3 | +/+ males n= 14; +/+ females n= 14; +/- males n= 13; +/- females n= 13 | n= 2 removed: mouse did not produce pellet (1); stool score missing (1) | p= 0.0004*** | Genotype p= 0.6109; Sex p= 0.9718 | Genotype x Sex p= 0.8782 | n/a | litter, cage |
| faecal water content | faecal water content (%) | 4D | 3 | +/+ males n= 9; +/+ females n= 14; +/- males n= 12; +/- females n= 13 | n= 8 removed: did not produce enough pellets for assay | p= 0.1220 | Genotype p= 0.7015 ; Sex p= 0.1449 | Genotype x Sex p= 0.7058 | n/a | litter, cage |
| faecal calprotectin | faecal calprotectin (pg/mL) | 4E | 3 | +/+ males n= 7; +/+ females n= 9; +/- males n= 9; +/- females n= 9 | n= 5 removed: high coefficient of variation (CV, %) for technical replicates | p= 2.18e-05 *** | Genotype p= 0.02397* ; Sex p= 0.0925 | Genotype x Sex p= 0.6912 | n/a | litter, cage |
| gut permeability | FITC-dextran (μg/mL) | 4F | 3 | +/+ males n= 10; +/+ females n= 12; +/- males n= 11; +/- females n= 8 | n= 2 removed: insufficient plasma volume for assay | p= 0.0332* | Genotype p= 0.2138 ; Sex p= 0.0052** | Genotype x Sex p= 0.0476* | Males +/+ vs +/-: p= 0.2138; females +/+ vs +/- p= 0.1210 | litter, cage, day of experiment |
| brain weight to body weight ratio | brain weight to body weight ratio | 4G | 3 | +/+ males n= 11; +/+ females n= 11; +/- males n= 12; +/- females n= 8 | none | p= 0.0001*** | Genotype p= 0.0016** ; Sex p= 2.2e-16 *** | Genotype x Sex p= 0.5541 | n/a | litter, cage |
| caecum weight to body weight ratio | caecum weight to body weight ratio | 4H | 3 | +/+ males n= 11; +/+ females n= 8; +/- males n= 10; +/- females n= 8 | n= 2 removed: cecum content leak during dissection | p= 0.2850 | Genotype p= 0.1091 ; Sex p= 0.2530 | Genotype x Sex p= 0.1930 | n/a | litter, cage |
| caecum length to body weight ratio | caecum length to body weight ratio | 4I | 3 | +/+ males n= 11; +/+ females n= 8; +/- males n= 10; +/- females n= 8 | n= 2 removed: cecum content leak during dissection | p= 0.0343 | Genotype p= 0.3465 ; Sex p= 4.899e-11 | Genotype x Sex p= 0.8707 | n/a | litter, cage |
| colon length to body weight ratio | colon length to body weight ratio | 4J | 3 | +/+ males n= 11; +/+ females n= 10; +/- males n= 9; +/- females n= 8 | n= 1 removed: damage to colon tissue during dissection | p= 0.0090** | Genotype p= 0.3636 ; Sex p= 1.95e-13 | Genotype x Sex p= 0.5065 | n/a | litter, cage |
| body weight | body weight (g) | 4K | 3 | +/+ males n= 14; +/+ females n= 14; +/- males n= 14; +/- females n= 14 | none | p= 5.694e-06 *** | Genotype p= 0.8425 ; Sex p= 2.2e-16***; Age p= 2.2e-16*** | Genotype x Sex p= 0.9594; Genotype x Age p= 0.0051**; Sex x Age p= 2.2e-16***; Genotype x Sex x Age p= 0.1679 | all relevant: n.s. | litter, cage |
| food consumption | food consumed (g/mouse/day) | 4L | 3 | (cages) +/+ males n= 6; +/+ females n= 6; +/- males n= 6; +/- females n= 6 | none | p= 0.3398 | Genotype p= 0.1294 ; Sex p= 0.0009*** | Genotype x Sex p= 0.3580 | n/a | n/a |
| gut microbiota alpha diversity | alpha diversity: Simpson’s dominance | 5A | 3 | +/+ males n= 7; +/+ females n= 8; +/- males n= 8; +/- females n= 8 | n= 1 removed: low read number in 16S sequencing | p= 3.29e-05*** | Genotype p= 0.2605*; Sex p= 0.9912 | Genotype x Sex p= 0.2921 | n/a | n/a |
| gut microbiota alpha diversity | alpha diversity: Shannon entropy | 5B | 3 | +/+ males n= 7; +/+ females n= 8; +/- males n= 8; +/- females n= 8 | n= 1 removed: low read number in 16S sequencing | p= 0.0250* | Genotype p= 0.0432*; Sex p= 0.8669 | Genotype x Sex p= 0.1301 | n/a | n/a |
| gut microbiota beta diversity | beta diversity: Aitchison distance over two principal components | 5C | 3 | +/+ males n= 7; +/+ females n= 8; +/- males n= 8; +/- females n= 8 | n= 1 removed: low read number in 16S sequencing | n/a | (PERMANOVA)Genotype p= 0.1020; Sex p= 0.0010** | Genotype x Sex p= 0.0240* | (pairwise PERMANOVA) males: +/+ vs +/- p= 0.0120* | n/a |
|  |  |  |  |  |  |  |  |  | females: +/+ vs +/- p= 0.3020 |  |
|  |  |  |  |  |  |  |  |  | +/+: males vs females p= 0.002** |  |
|  |  |  |  |  |  |  |  |  | +/-: males vs females p= 0.0192* |  |
| gut microbiota species abundance | relative abundance | 5E | 3 | +/+ males n= 7; +/+ females n= 8; +/- males n= 8; +/- females n= 8 | n= 1 removed: low read number in 16S sequencing | n/a | Males and females analysed separately. | n/a | n/a | n/a |
|  |  |  |  |  |  |  | Males: Anaerotaenia torta \| Genotype q=0.0012**; Clostridium sp SY8519 \| q=0.028*; [Clostridium] polysaccharolyticum \| q=0.0352*; Blautia producta \| q=0.0093**; Neglecta timonensis \| q=0.0081**; Mordavella massiliensis \| q=0.0122*; all other species n.s.; |  |  |  |
|  |  |  |  |  |  |  | Females: all species n.s. |  |  |  |
| brain vs alpha diversity correlation | brain to body weight ratio vs Simpson’s dominance | 5F | 3 | +/+ males n= 4; +/+ females n= 6; +/- males n= 7; +/- females n= 3 | n= 11 removed: brain weight not measured | n/a | R=0.3156 \| R2=0.09647 \| p= 0.0552 | n/a | n/a | n/a |
| predicted functional pathways beta diversity | beta diversity: Aitchison distance over two principal components | 6A | 3 | +/+ males n= 7; +/+ females n= 8; +/- males n= 8; +/- females n= 8 | n= 1 removed: low read number in 16S sequencing | n/a | (PERMANOVA) Genotype p= 0.5840; Sex p= 0.0230* | Genotype x Sex p= 0.3690 | (pairwise PERMANOVA) males: +/+ vs +/- p= 0.444 | n/a |
|  |  |  |  |  |  |  |  |  | females: +/+ vs +/- p= 0.6990 |  |
|  |  |  |  |  |  |  |  |  | +/+: males vs females p= 0.4380 |  |
|  |  |  |  |  |  |  |  |  | +/-: males vs females p= 0.4380 |  |
| predicted functional pathways abundance | relative abundance | analysis not graphically represented, data shown in 6B | 3 | +/+ males n= 7; +/+ females n= 8; +/- males n= 8; +/- females n= 8 | n= 1 removed: low read number in 16S sequencing | n/a | Males and females analysed separately (Maaslin2). | n/a | n/a | n/a |
|  |  |  |  |  |  |  | Males: PWY-6545 \| Genotype: q= 0.0091** |  |  |  |
|  |  |  |  |  |  |  | Females: PWY-6545 \| Genotype: q= 0.0323* |  |  |  |
| predicted functional pathways abundance | relative abundance: pairwise Wilcoxon rank-sum to determine differences between groups | 6B | 3 | +/+ males n= 7; +/+ females n= 8; +/- males n= 8; +/- females n= 8 | n= 1 removed: low read number in 16S sequencing | n/a | n/a | n/a | males: +/+ vs +/- p= 0.0420* | n/a |
|  |  |  |  |  |  |  |  |  | females: +/+ vs +/- p= 0.0150* |  |
|  |  |  |  |  |  |  |  |  | +/+: males vs females p= 0.0070** |  |
|  |  |  |  |  |  |  |  |  | +/-: males vs females p= 0.4420 |  |
| supervised integration analysis | correlation between microbial species and brain weight | 6F | 3 | males only: +/+ n= 4; +/- n=7 | excluded individuals where all relevant measurements were not available (as determined in preliminary analysis) | n/a | Clostridium sp. SY8519 \| r = 0.8549281; Anaerotaenia torta \| r = 0.8368198; Clostridium polysaccharolyticum \| r = 0.8023390; Blautia sp. SC05B48 \|r = 0.7662035; Mordavella massiliensis \| r = -0.7610691; Neglecta timonensis \|r = -0.7355181; Bacteroides pectinophilus \|r = -0.6916692. | n/a | n/a | n/a |
| large open field | time spent in centre (%) | S1A | 2 | +/+ males n= 14; +/+ females n= 13; +/- males n= 14; +/- females n= 14 | none | p= 0.0056** | Genotype p= 0.5320 ; Sex p= 0.7477 | Genotype x Sex p= 0.3702 | n/a | litter, cage |
| light-dark box | time in light zone (%) | S1B | 2 | +/+ males n= 14; +/+ females n= 13; +/- males n= 14; +/- females n= 14 | none | p= 0.7706 | Genotype p= 0.1807 ; Sex p= 0.9816 | Genotype x Sex p= 0.6270 | n/a | litter, cage |
| elevated plus maze | time in open arms (%) | S1C | 2 | +/+ males n= 14; +/+ females n= 13; +/- males n= 14; +/- females n= 14 | none | p= 0.7052 | Genotype p= 0.5014 ; Sex p= 0.5329 | Genotype x Sex p= 0.4433 | n/a | litter, cage |
| novelty-suppressed feeding test | latency to feed (s) | S1D | 2 | +/+ males n= 14; +/+ females n= 13; +/- males n= 14; +/- females n= 14 | none | p= 0.0005*** | Genotype p= 0.3040; Sex p= 0.7800 | Genotype x Sex p= 0.2110 | n/a | litter, cage |
| novelty-suppressed feeding test | body weight lost (%) | S1E | 2 | +/+ males n= 14; +/+ females n= 13; +/- males n= 14; +/- females n= 14 | none | p= 0.8767 | Genotype p= 0.3753 ; Sex p= 0.0459* | Genotype x Sex p= 0.8122 | n/a | litter, cage |
| novelty-suppressed feeding test | food consumed (g) | S1F | 2 | +/+ males n= 14; +/+ females n= 13; +/- males n= 14; +/- females n= 14 | none | p= 0.4746 | Genotype p= 0.0222* ; Sex p= 0.3835 | Genotype x Sex p= 0.6279 | n/a | litter, cage |
| saccharin preference test | saccharin preference (%) | S1G | 2 | +/+ males n= 14; +/+ females n= 13; +/- males n= 14; +/- females n= 14 | none | p= 3.091e-8 *** | Genotype p= 0.8005 ; Sex p= 0.6786; Day p= 0.1909 | Genotype x Sex p= 0.7860; Genotype x Day p= 0.0859; Sex x Day p= 0.1673; Genotype x Sex x Day p= 0.2574 | n/a | litter, cage |
| Porsolt swim test | immobility (%) | S1H | 2 | +/+ males n= 14; +/+ females n= 13; +/- males n= 14; +/- females n= 14 | none | p= 0.3149 | Genotype p= 0.4012; Sex p= 0.7081 | Genotype x Sex p= 0.4310 | n/a | litter, cage |
| Porsolt swim test | latency to float (s) | S1I | 2 | +/+ males n= 14; +/+ females n= 13; +/- males n= 14; +/- females n= 14 | none | p= 3.848e-10*** | Genotype p= 0.3886; Sex p= 0.7596 | Genotype x Sex p= 0.0951 | n/a | litter, cage |
| marble burying test | total marbles buried | S1J | 1 | +/+ males n= 9; +/+ females n= 14; +/- males n= 13; +/- females n= 13 | n= 6 removed: climbed walls of arena | p= 0.0231* | Genotype p= 0.7021; Sex p= 0.0708 | Genotype x Sex p= 0.1728 | n/a | litter, cage |
| large open field | total distance travelled (mm) | S2A | 2 | +/+ males n= 14; +/+ females n= 13; +/- males n= 14; +/- females n= 14 | none | p= 0.0869 | Genotype p= 0.6359; Sex p= 0.2510 | Genotype x Sex p= 0.7595 | n/a | litter, cage |
| light-dark box | total distance travelled (cm) | S2B | 2 | +/+ males n= 14; +/+ females n= 13; +/- males n= 14; +/- females n= 14 | none | p= 0.3481 | Genotype p= 0.1236; Sex p= 0.4050 | Genotype x Sex p= 0.2496 | n/a | litter, cage |
| novel object recognition test | total distance travelled (mm) | S2C | 1 | +/+ males n= 14; +/+ females n= 10; +/- males n= 14; +/- females n= 13 | n= 4 removed: climbed onto objects | p= 0.0044** | Genotype p= 0.1236; Sex p= 0.4050 | Genotype x Sex p= 0.2496 | n/a | litter, cage |
| social interaction test | total distance travelled (mm) | S2D | 2 | +/+ males n= 14; +/+ females n= 13; +/- males n= 14; +/- females n= 14 | none | p= 0.0190* | Genotype p= 0.9644; Sex p= 0.0860 | Genotype x Sex p= 0.1130 | n/a | litter, cage |
| rotarod | average latency to fall (s) | S2E | 2 | +/+ males n= 14; +/+ females n= 13; +/- males n= 14; +/- females n= 14 | none | p= 0.3094 | Genotype p= 0.4648; Sex p= 0.0960 | Genotype x Sex p= 0.4665 | n/a | litter, cage |
| caecum weight to body weight ratio | caecum weight to body weight ratio | S3A | 2 | +/+ males n= 14; +/+ females n= 13; +/- males n= 14; +/- females n= 14 | damage to caecum, excluded 1 +/+ female | p= 0.0611 | Genotype p= 0.8839; Sex p< 0.001*** | Genotype x Sex p= 0.5554 | n/a | litter, cage |
| caecum length to body weight ratio | caecum length to body weight ratio | S3B | 2 | +/+ males n= 14; +/+ females n= 12; +/- males n= 14; +/- females n= 14 | damage to caecum, excluded 1 +/+ female | p= 0.0006*** | Genotype p= 0.1375; Sex p= 0.0002*** | Genotype x Sex p= 0.3013 | n/a | litter, cage |
| colon length to body weight ratio | colon length to body weight ratio | S3C | 2 | +/+ males n= 14; +/+ females n= 13; +/- males n= 14; +/- females n= 14 | none | p= 0.0128* | Genotype p= 0.1536; Sex p< 0.0001*** | Genotype x Sex p= 0.7336 | n/a | litter, cage |

**Supplementary table 2.** This table contains exact statistics and details about statistical analyses for all main figures, including *p* values and sample sizes for each analysis. Results of pairwise comparisons were only included where interactions with the Genotype factor were significant. Where Genotype x Sex was significant, all comparisons relevant to +/+ and +/- subgroup comparisons are listed. Where additional factors (Age, Block, etc) were involved, only selected relevant comparisons are listed due to space limitations, including where a comparison between +/- and +/+ subgroups was significant (α< 0.05) or indicated a statistical trend (α< 0.09).
